# Chronic ER Stress Triggers Cell‐Surface Chaperones as the Therapeutic Targets of CAR Cells in Acute Myeloid Leukemia

**DOI:** 10.1002/advs.202511573

**Published:** 2025-10-23

**Authors:** Yimin Zhou, Zhenfei Zhong, Peng Hu, Weigang Wang, Ying Song, Na Yang, Fangyan He, Yajie Li, Qi Sa, Yanmei Yang, Qinmiao Sun, Tonghua Yang, Beibei Zhang, Dahua Chen

**Affiliations:** ^1^ Institute of Biomedical Research Yunnan University Kunming Yunnan 650500 China; ^2^ Department of Hematology The First People's Hospital of Yunnan Province Kunming Yunnan 650032 China; ^3^ Yunnan Provincial Clinical Medical Center for Blood Diseases and Thrombosis Prevention and Treatment Kunming Yunnan 650032 China; ^4^ State Key Laboratory of Organ Regeneration and Reconstruction Institute of Zoology Chinese Academy of Sciences Beijing 100101 China; ^5^ Beijing Institute for Stem Cell and Regenerative Medicine Beijing 100101 China; ^6^ School of Life Sciences University of Chinese Academy of Sciences Beijing 100049 China; ^7^ Southwest United Graduate School Kunming Yunnan 650500 China

**Keywords:** acute myeloid leukemia, cell surface chaperone, chimeric antigen receptor, chronic ER stress, immunotherapy, natural killer cell

## Abstract

Acute myeloid leukemia (AML) is a heterogeneous malignancy with low survival rates, primarily due to its inherent complexity. This underscores the urgent need to identify specific targets for precision medicine. Here, multi‐omics approaches are utilized and discover that AML cells undergo chaperone‐mediated chronic endoplasmic reticulum (ER) stress. Through integrative analyses of single‐cell RNA‐seq, cell‐surface proteomes, and cellular biology, ER chaperone proteins (e.g., HSP90B1 and P4HB) are identified as potential neoantigens that translocate to the cell surface upon chronic ER stress. These results suggest that these proteins, especially in FLT3‐ITD^+^ AML cells, show great promise as diagnostic markers and therapeutic targets. To explore the therapeutic potential, chimeric antigen receptor–natural killer (CAR‐NK) cells targeting surface‐localized HSP90B1 are engineered. These engineered cells show selective cytotoxicity both in vitro and in animal models. This study not only identifies neoantigens as specific biomarkers refining AML classification, but also emphasizes the potential of immunotherapy‐based precision treatments for AML.

## Graphical abstract

Acute myeloid leukemia (AML) remains a therapeutic challenge due to its heterogeneity and limited targets. Here, multi‐omics analyses are utilized, and it is revealed that AML cells, particularly the FLT3‐ITD^+^ subtype, undergo chaperone‐mediated ER stress, inducing surface translocation of ER chaperones. Subsequently, CAR‐NK cells targeting these chaperone proteins are engineered, demonstrating specific anti‐leukemia efficacy in vitro and in vivo, which offers a novel immunotherapeutic strategy for precision AML treatment.

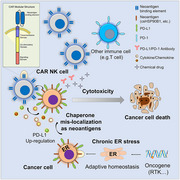

## 1 Introduction

Acute myeloid leukemia (AML) is a complex and aggressive disease characterized by the clonal proliferation of myeloid progenitor cells in the bone marrow.^[^ ^1^, ^2^ ^]^ In adults, AML represents the most prevalent form of acute leukemia. Driven by the malignancy of hematopoietic stem or progenitor cells (HSPCs), its progression is rapid, mandating prompt diagnosis and treatment.^[^ ^3^, ^4^ ^]^ Fundamental therapeutic approaches have largely remained unchanged for decades. The standard treatment typically involves an induction phase of intensive chemotherapy, often following the “3+7” regimen.^[^ ^5^ ^]^ Recently, considerable efforts have been made to improve treatment strategies such as tyrosine kinase inhibitors (TKIs) and BCL‐2 inhibitors.^[^ ^6^, ^7^, ^8^, ^9^ ^]^ Nevertheless, many patients still experience relapse and develop drug resistance even after achieving initial complete responses.^[^ ^10^ ^]^ Thus, the classification and treatment of AML subtypes have transitioned from a primarily morphologic framework to one informed by molecular, genetic, and drug‐response profiles.^[^ ^1^, ^11^, ^12^ ^]^ This evolution highlights the urgent need for innovative precision therapy and mechanism understanding to address the complexity of AML and enhance patient outcomes.

Oncogenic stress, stemming from the continuous activity of various endogenous mutagenic accumulations, is implicated in diverse cancer processes.^[^ ^13^, ^14^ ^]^ Under stress conditions, malignant cells frequently attempt to restore cellular homeostasis for survival by initiating aberrant signaling pathways, degrading misfolded proteins, and performing specialized protein modifications like glycosylation, facilitated by a unique protein quality control (PQC) system.^[^ ^15^, ^16^ ^]^ During these processes, the synthesis of multiple chaperones is also induced, which sustains the stability of the intracellular cancer environment and fosters cancer cell survival.^[^ ^17^ ^]^ Moreover, tumor‐associated antigens (TAAs), tumor‐specific antigens (TSAs), or neoantigens emerge as a consequence of the adaptive oncogenic stress induced by genomic mutations in tumor cells.^[^ ^14^, ^18^, ^19^, ^20^ ^]^ These critical adaptive cell stress responses not only fuel disease progression but also impact the efficacy of drug therapies.^[^ ^13^, ^21^, ^22^ ^]^ When the cells fail to regain balance after treatment, the response shifts from the pro‐survival “adaptive state” to the pro‐apoptotic “terminal state.”^[^ ^13^ ^]^ Thus, these mechanistic insights and associated novel targets offer valuable guidance for potential therapeutic interventions.^[^ ^17^, ^21^, ^22^ ^]^

Chimeric antigen receptor (CAR) cell therapy represents a transformative advancement in cancer treatment.^[^ ^23^ ^]^ Genetically engineered T lymphocytes expressing a CAR targeting the antigens such as CD19 or BCMA have demonstrated remarkable success in treating acute lymphoblastic leukemia (ALL) and diffuse large B‐cell lymphoma (DLBCL).^[^ ^24^, ^25^, ^26^ ^]^ Extending CAR cell therapy to AML poses significant challenges due to the limited availability of AML‐specific antigens. Surface antigens commonly expressed by AML blasts, including CD123,^[^ ^27^ ^]^ CD33,^[^ ^28^ ^]^ CD38,^[^ ^29^ ^]^ CLL‐1,^[^ ^30^ ^]^ or FLT3,^[^ ^31^ ^]^ are also present on normal myeloid precursors, restricting their use as exclusive therapeutic targets. Moreover, ongoing preclinical and clinical studies are investigating additional potential targets, such as lymphocyte‐associated receptors (e.g., CD4^[^ ^32^ ^]^ and CD7^[^ ^33^ ^]^) and immune regulatory ligands (e.g., PD‐L1,^[^ ^34^ ^]^ CD70,^[^ ^35^ ^]^ and NKG2DL^[^ ^36^ ^]^). Comprehensive mass spectrometry (MS) and related technologies have been instrumental in identifying novel targets for cancer drug development,^[^ ^37^ ^]^ which has significantly enhanced our understanding of the surfaceome and holds promise for discovering neoantigens for immunotherapy development.

In this study, we performed multi‐omics analyses and identified several chaperone proteins that are normally localized to the endoplasmic reticulum (ER) but translocate to the cell surface across various AML subtypes, including FLT3‐ITD^+^ AML cells. Mechanistically, oncogenic stress induced by mutations such as FLT3 was found to trigger ER protein translocation to the cell surface through signal integration with aberrant gene expression. In addition, we developed CAR‐natural killer (NK) cells targeting the cell‐surface protein HSP90B1 (csHSP90B1), demonstrating their potent ability to eliminate AML cells without causing significant toxicity, which provides a novel avenue for eradicating malignant cells capable of enduring chronic oncogenic stress. Collectively, our findings introduce a promising strategy for advancing AML immunotherapy.

## 2 Results

### 2.1 Cell‐Surface Proteomic Analysis Reveals Potential Neoantigens of AML Cells

**Figure 1:**
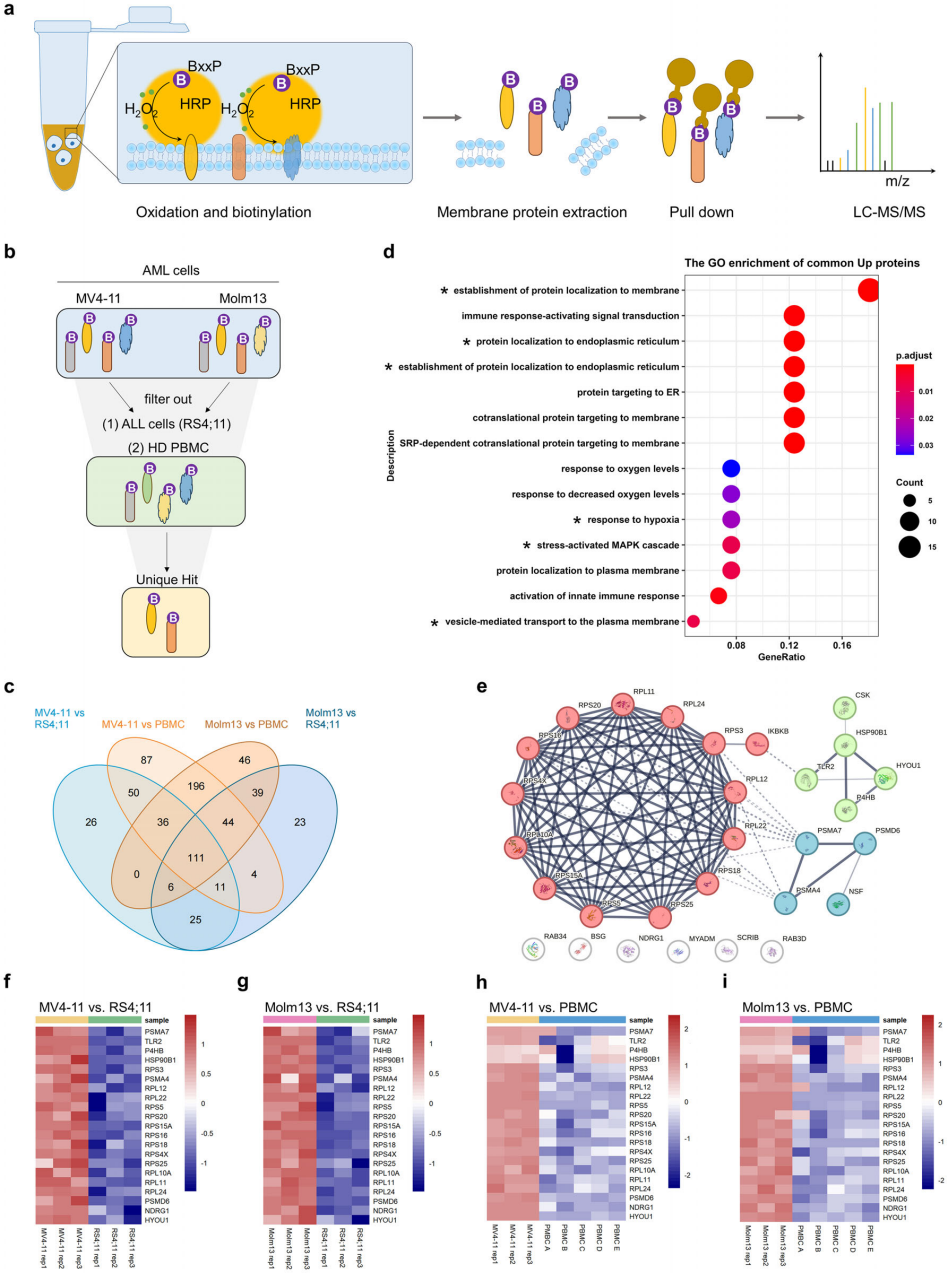
Cell‐surface proteomic approach for the identification of potential AML neoantigens. a) Schematic of the biotinylation principle for labeling cell‐surface proteins in a mass spectrometry experiment. b) Workflow illustrating the cell‐surface labeling assay used to identify unique cell‐surface proteins in AML cells through mass spectrometry analysis. c) Venn diagram depicting the overlap of cell‐surface proteins identified in AML (MV4‐11 and Molm13), non‐AML (RS4;11), and PBMC proteomic datasets. d) Gene ontology analysis indicating significant enrichment of AML‐specific cell‐surface proteins in stress‐related biological processes. e) STRING protein interaction network analysis showing interactions among selected cell‐surface proteins. f–i) Heatmaps displaying the expression patterns of selected cell‐surface proteins in various comparative analyses.

Cell‐surface proteins play a critical role in regulating various cellular functions at the interface of cells and their environment. In cancer cells, neoantigens are frequently produced due to mutations, oncogenic stresses, or abnormal protein expression on the cell surface, making them potential targets for therapeutic development. In AML, the current clinical therapeutic efficacy is unsatisfactory due to disease complexity and a lack of specific targets, urgently necessitating precision therapies. To search for the novel targets, we employed cell‐surface protein labeling assays in multiple AML cancer cell lines, including MV4‐11 and Molm13, a non‐AML cancer cell line (RS4;11, an ALL cell line) and peripheral blood mononuclear cells (PBMCs) collected from healthy donors (HDs) as controls. Cell‐surface protein labeling assays were conducted following previously established methods.^[^ ^38^, ^39^, ^40^, ^41^, ^42^ ^]^ These assays utilized the biotin‐XX‐phenol (BxxP) probe as the labeling agent, with horseradish peroxidase (HRP) catalyzing protein biotinylation in the presence of H_2_O_2_ (**Figure** **1**a). To evaluate the labeling specificity and efficacy, we performed immunofluorescence and immunoblot analysis on biotinylated samples. As anticipated, immunofluorescence analysis demonstrated marked biotinylation of cell‐surface and plasma membrane‐associated proteins (Figure S1a, Supporting Information). Subsequent immunoblot analysis revealed that the Bxxp reagent exhibited robust biotinylation activity toward a wide range of proteins, significantly exceeding the endogenously biotinylated proteins in control reactions conducted in the absence of either HRP or H_2_O_2_ (Figure S1b, Supporting Information). As for proteomic analysis, biotinylated proteins were purified using streptavidin beads and analyzed through MS. Background controls, lacking either HRP or H_2_O_2_, were included to address nonspecific binding and endogenous biotinylation. The analysis generated extensive cell‐surface proteomic datasets, including 993, 960, 925, and 932 proteins from MV4‐11, Molm13, RS4;11, and PBMC samples, respectively (Figure S1c and Dataset S1, Supporting Information). To identify AML‐specific surface proteins, we compared the proteomic datasets of AML cells (MV4‐11 and Molm13) with those of RS4;11 cells. This comparison revealed the presence of 265 and 263 proteins at higher levels on the surfaces of MV4‐11 and Molm13 cells, respectively (Figure S1d,e and Dataset S2, Supporting Information). A subsequent comparison between AML cells and healthy PBMCs identified 539 and 478 proteins on MV4‐11 and Molm13 cells, respectively (Figure S1f,g and Dataset S2, Supporting Information). Notably, this analysis highlighted several proteins previously recognized as potential AML targets, such as CD33^[^ ^43^ ^]^ and IL1RAP.^[^ ^44^, ^45^ ^]^ (Figure S1d–g, Supporting Information). Further comparative analyses (Figure 1b) revealed 111 cell‐surface proteins specifically enriched on MV4‐11 and Molm13 cells (Figure 1c). Gene Ontology (GO) analysis identified enrichment of stress‐related biological processes among these proteins, notably establishment of protein localization to the membrane, endoplasmic reticulum (ER) protein localization, response to hypoxia, and stress‐activated MAPK cascade (Figure 1d; Figure S1h, Supporting Information). STRING network analysis further corroborated the functional interplay of proteins linked to ER protein localization, plasma membrane protein targeting, and hypoxia response (Figure 1e). Heatmap profiling demonstrated preferential surface expression of ER‐localized proteins and hypoxia‐responsive proteins on MV4‐11 and Molm13 AML cells compared to RS4;11 cells and PBMCs (Figure 1f–i). Taken together, these findings suggest that AML cells, particularly MV4‐11 and Molm13, exhibit a distinct surface proteomic signature characterized by elevated or specific expression of ER‐associated proteins, implicating stress adaptation mechanisms in their biology.

### 2.2 FLT3‐ITD^+^ Mutation‐Induced Oncogenic Stress Triggers the Translocation of ER Proteins to the Cell Surface

**Figure 2:**
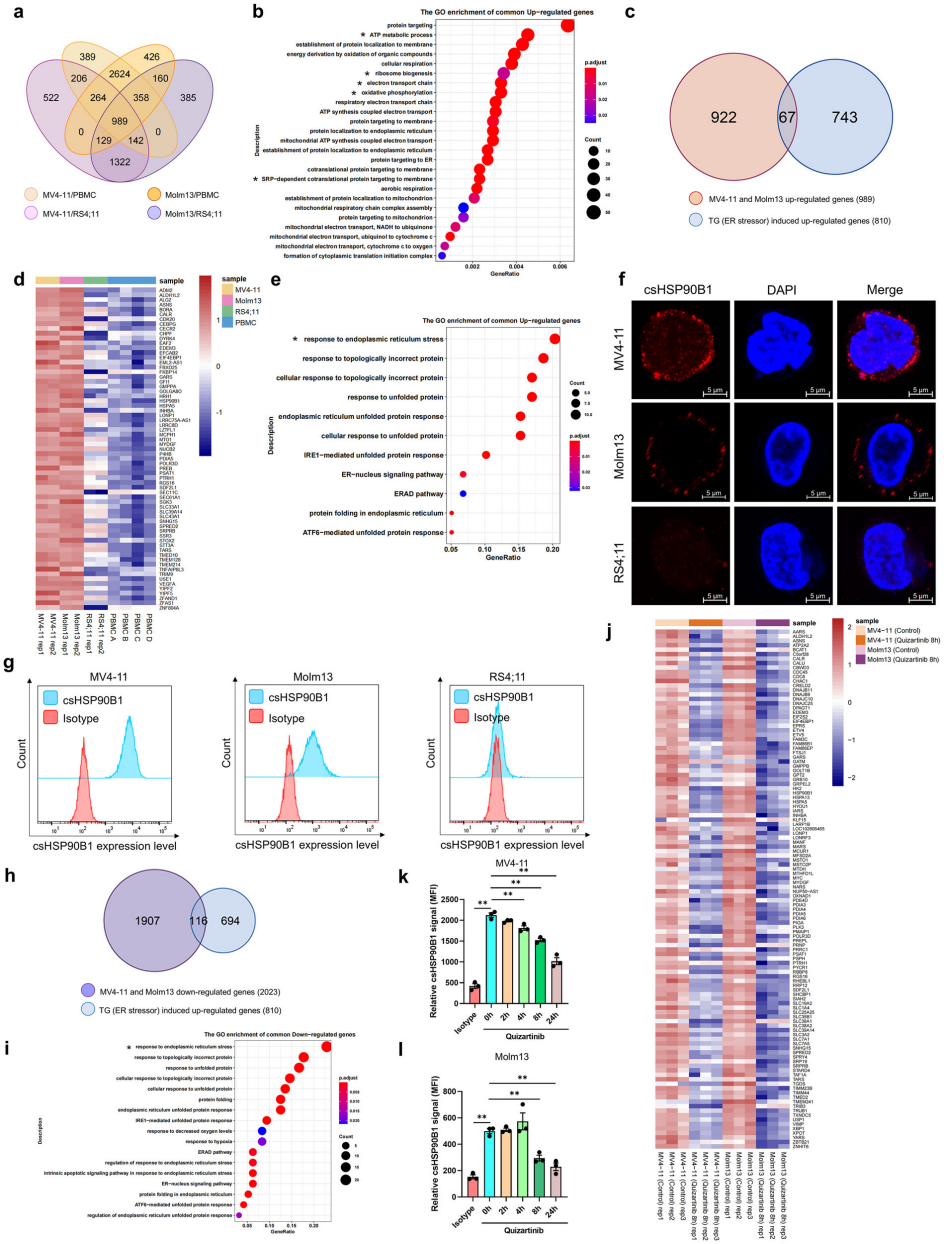
Oncogenic stress induced by FLT3‐ITD^+^ mutation is closely associated with the translocation of ER proteins to the cell surface. a) Venn diagram illustrating upregulated transcription profiles across comparisons, including MV4‐11 versus RS4;11, Molm13 versus RS4;11, MV4‐11 versus PBMC, and Molm13 versus PBMC. b) Gene ontology (GO) analysis of 989 commonly upregulated genes in MV4‐11 and Molm13 cells. c) Venn diagram comparing 989 commonly upregulated AML genes (MV4‐11 and Molm13) with 810 ER stressor (thapsigargin, TG)‐induced upregulated genes in HEK293‐T cells. d) Heatmap displaying transcription profiles of 67 overlapping genes identified in the Venn diagram (c). e) GO analysis of the transcription profiles for the 67 overlapping genes in (d). f) Immunofluorescence images showing the cell‐surface protein HSP90B1 (csHSP90B1) in non‐permeabilized MV4‐11, Molm13, and RS4;11 cells. g) Flow cytometry analysis of csHSP90B1 expression on living MV4‐11, Molm13, and RS4;11 cells. h) Venn diagram illustrating the overlap between 2023 commonly downregulated genes (MV4‐11 and Molm13) following quizartinib treatment (100 nm, 8 h) and 810 TG‐induced upregulated genes in HEK293‐T cells. i) GO analysis of overlapping genes identified in the Venn diagram (h). j) Heatmap of genes associated with the ER stress response downregulated after quizartinib treatment (100 nm, 8 h). k,l) Quantification of csHSP90B1 expression levels in living MV4‐11 (k) and Molm13 (l) cells after quizartinib treatment (100 nm) at different time points, as analyzed by flow cytometry. Data represented as mean ± SEM of fluorescence intensity (MFI), *p*‐values are calculated using Student's *t*‐test, *n* = 3, **p < 0.01.

Based on the observation of the expression of ER‐related proteins, particularly ER chaperones such as HYOU1, HSP90B1, P4HB on the cell surface, we hypothesized that the AML cells analyzed in this study experience chronic ER stress. To verify this hypothesis, we generated multiple RNA‐seq datasets from MV4‐11, Molm13, RS4;11, and PBMCs of healthy donors. As a control for ER stress conditions, RNA‐seq datasets were generated from HEK293‐T cells treated with thapsigargin (TG), an ER stress inducer (Figure S2a–c, Supporting Information). Comprehensive RNA‐seq analysis revealed that compared with RS4;11 and PBMCs, many upregulated genes in MV4‐11 and Molm13 cells were associated with key cancer‐related biological processes, including “ATP metabolic process,” “ribosome biogenesis,” “electron transport chain,” “oxidative phosphorylation,” and “SRP‐dependent cotranslational protein targeting to membrane” (**Figure** **2**a,b; Figure S2d–g, Supporting Information). We also validated several key genes by RT‐qPCR, confirming the transcriptomic results (Figure S3a,b, Supporting Information). Furthermore, a substantial number of upregulated transcripts in MV4‐11 and Molm13 cells overlapped with TG‐induced ER stress‐related transcripts (Figure 2c–e). These results indicated that AML cells, specifically MV4‐11 and Molm13, endure chronic ER stress, which may drive the surface expression of ER chaperones such as HSP90B1 and P4HB, thereby supporting tumor survival. Previous studies have demonstrated that HSP90B1 acts as an HSP90‐like chaperone within the ER lumen, playing a critical role in the proper folding of client proteins.^[^ ^17^, ^46^ ^]^ Similarly, P4HB functions as an ER chaperone involved in regulating reactive oxygen species production and mitigating ER stress and protein misfolding.^[^ ^47^ ^]^ Recognizing the importance of these proteins, we performed immunostaining assays to confirm their presence on the cell surface. Signals for cell‐surface HSP90B1 (csHSP90B1) and P4HB (csP4HB) were reliably detected on AML cells, such as MV4‐11 and Molm13, but were absent on RS4;11 cells (Figure 2f; Figure S2h, Supporting Information). The antibody specificity and staining reliability for the important targets, like HSP90B1, were also confirmed (Figure S4, Supporting Information). Moreover, flow cytometry assays yielded consistent results, further validating the surface localization of csHSP90B1 and csP4HB on living MV4‐11 and Molm13 cells (Figure 2g; Figure S2i, Supporting Information). By contrast, csCD19 signals were observed on RS4;11 cells but not on AML cells, including MV4‐11 and Molm13 (Figure S2j,k, Supporting Information). Of note, a previous study has reported that GRP78 (HSPA5) protein was presented on the cell surface of AML cells;^[^ ^48^ ^]^ however, we found this protein was also detected on the surface of RS4;11 cells (Figure S1d,e, Supporting Information), suggesting that GRP78 may not be the unique cell‐surface marker for AML cells. Collectively, our findings indicate that certain chaperones, like csHSP90B1 are selectively expressed on the surface of AML cells, suggesting their potential significance in tumor biology.

Having observed that MV4‐11 and Molm13 cells endure aberrant ER stress response, and given that both types of these cells harbor FLT3‐ITD^+^ mutation, we sought to ask whether the aberrant ER stress response and cell‐surface expression of HSP90B1 and P4HB could be attributed to this mutation. To address this issue, we treated MV4‐11 and Molm13 cells with quizartinib, a tyrosine kinase inhibitor (TKI) known to suppress FLT3‐ITD^+^ mutant kinase phosphorylation and activity.^[^ ^49^ ^]^ RNA‐seq analyses and RT‐qPCR experimental validations revealed that TKI treatment significantly reduced the aberrant response of ER stress in these cells, as indicated by the downregulation of numerous stress‐related genes in those cells (Figure 2h–j; Figures S3c and S5a–c, Supporting Information). Attempts to knock out FLT3 in FLT3‐ITD⁺ cell lines (MV4‐11 and Molm13) were unsuccessful, likely because FLT3‐ITD^+^ mutation acts as an oncogenic driver essential for cell survival. Notably, treatment with tyrosine kinase inhibitors (TKIs) reduced the viability of these FLT3‐ITD⁺ cells and simultaneously decreased the expression of csHSP90B1 and csP4HB. Flow cytometry assays showed a marked reduction in csHSP90B1 and csP4HB signals following TKI treatment in the rest surviving leukemic cells of both MV4‐11 and Molm13 cells. These findings suggested that constitutive kinase activity of FLT3‐ITD^+^ mutation serves as an important contributor to the regulation of the cell‐surface localization of chaperones, including HSP90B1 and P4HB (Figure 2k,l; Figure S5d,e, Supporting Information). To investigate whether overexpression of FLT3‐ITD^+^ mutation alone is sufficient to induce csHSP90B1 and csP4HB expression, we generated BaF3 cell lines overexpressing either FLT3‐ITD^+^ or wild‐type FLT3 proteins. BaF3 cells are murine pro‐B cell models widely used for studying oncogenic tyrosine kinases.^[^ ^49^, ^50^ ^]^ Flow cytometry analysis revealed significantly higher levels of csHSP90B1 and csP4HB in FLT3‐ITD^+^ overexpressing cells compared to those expressing wild‐type FLT3 (Figure S5f–i, Supporting Information). Overall, those results indicate that FLT3‐ITD^+^ mutation in the AML cells likely induces the aberrant ER stress response and the cell‐surface expression of certain chaperones. In this study, we demonstrate that FLT3‐ITD^+^ mutation‐driven oncogenic stress induces chronic ER stress in AML cells, promoting chaperone surface translocation, including HSP90B1. Additionally, pharmacologic suppression of FLT3‐ITD^+^ mutation signaling via TKIs markedly reduced chaperone surface translocation, which was associated with attenuation of ER stress in the leukemic cells (Figure 2h–l; Figure S5a–e, Supporting Information). Particularly, as established by Tsai et al. in HeLa cervical carcinoma cells, ER stress activates IRE1α, triggering a SRC tyrosine kinase‐mediated signaling cascade that drives ER chaperone re‐localization to the plasma membrane.^[^ ^51^ ^]^ Previous studies have illustrated that SRC is a downstream signaling mediator in FLT3‐ITD^+^ AML cells.^[^ ^52^ ^]^ Here, we further performed flow cytometry analyses, which revealed that pharmacological inhibition of IRE1α or SRC significantly reduced surface chaperone exposure (Figure S6, Supporting Information). Collectively, these findings establish FLT3‐ITD^+^ mutation‐induced ER stress mediated through the IRE1α‐SRC signaling axis as a mechanistic driver of chaperone surface translocation in AML.

### 2.3 Malignant AML Primary Cells Endure Chronic ER Stress and Display AML‐Associated Neoantigens

**Figure 3:**
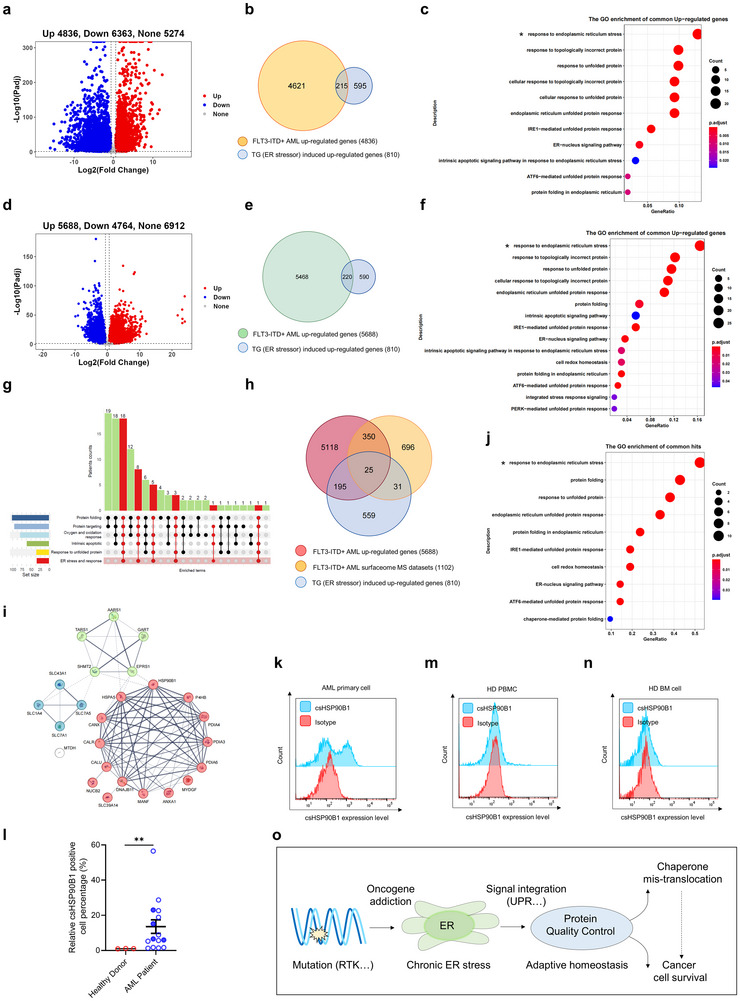
Cell‐surface chaperones serve as potential immunotherapeutic targets impersonating novel AML‐associated neoantigens. a) Volcano plot showing transcription profiles of FLT3‐ITD^+^ AML cells compared with peripheral blood mononuclear cells (PBMCs) from healthy individuals. b) Venn diagram illustrating the overlap between 4836 upregulated genes in FLT3‐ITD^+^ AML and 810 upregulated genes induced by the ER stressor thapsigargin (TG) in HEK293‐T cells. c) Gene ontology (GO) analysis of overlapping genes identified in the Venn diagram (b). d) Volcano plot of transcription profiles from 131 cases of FLT3‐ITD^+^ AML patient cells compared with PBMCs from healthy individuals. e) Venn diagram depicting the overlap between 5688 upregulated genes in 131 cases of FLT3‐ITD^+^ AML and 810 TG‐induced upregulated genes in HEK293‐T cells. f) GO analysis of overlapping genes identified in the Venn diagram (e). g) Summary of shared aberrant biological processes among 112 comparable FLT3‐ITD^+^ AML cases. h) Venn diagram illustrating overlapping hits among upregulated transcription profiles from 131 cases of FLT3‐ITD^+^ AML, mass spectrometry datasets from six FLT3‐ITD^+^ AML cases (included if proteins were detectable in more than two cases), and HEK293‐T cells with upregulated genes induced by TG. i) STRING protein interaction network analysis of 25 overlapping hits identified in the Venn diagram (h). j) GO analysis of the 25 overlapping hits from the Venn diagram (h). k) Representative flow cytometry analysis showing csHSP90B1 expression in primary cells from an AML patient. l) Quantitative percentage analysis of subpopulation with prominent csHSP90B1 signals in samples from 15 AML patients and 3 normal bone marrow samples from healthy donors assessed using flow cytometry; dark blue points indicate FLT3‐ITD^+^ AML cases. Data represented as mean ± SEM, *p*‐value is calculated using Student's *t*‐test, *n* = 3 in the healthy donor group and *n* = 15 in the AML patient group, **p < 0.01. m,n) Flow cytometry analysis of csHSP90B1 expression in healthy PBMCs (m) and healthy bone marrow cells (n). o) Working model of how FLT3‐ITD^+^ mutation induces translocation of ER proteins to the cell surface via adaptive ER stress homeostasis, highlighting their potential as therapeutic targets for cancer.

Furthermore, we analyzed primary samples from FLT3‐ITD^+^ AML patients. RNA‐seq analysis of these samples revealed significant upregulation of ER stress‐induced transcripts in AML blast cells compared with PBMCs from healthy donors, supporting the hypothesis that these cells experience chronic ER stress (**Figure** **3**a–c). To strengthen these findings, we reanalyzed clinical data from public databases,^[^ ^37^ ^]^ including 131 FLT3‐ITD^+^ AML patients. The results consistently indicated that FLT3‐ITD^+^ AML cells exhibited signs of chronic ER stress (Figure 3d–f; Figure S7a–d, Supporting Information). Specifically, GO analyses of 112 comparable cases highlighted shared characteristics, including aberrant protein folding, oxygen and oxidative stress responses, and persistent ER stress (Figure 3g; Dataset S3, Supporting Information). Integrative analyses of cell‐surface proteomic data from MS, upregulated genes from RNA‐seq datasets in FLT3‐ITD^+^ AML samples, and ER stress‐induced upregulated genes in HEK293‐T cells further validated these findings (Figure 3h). These analyses demonstrated a significant enrichment of GO terms related to “response to ER stress,” which encompassed specific chaperones, including HSP90B1, HSPA5, P4HB, CANX, and CALR (Figure 3h–j).

Recent studies have documented certain chaperones as promising targets for cancer therapy.^[^ ^13^, ^53^, ^54^, ^55^ ^]^ For instance, surface‐expressed P4HB has been identified in various malignant and normal cell types, including leukemic cells, vascular smooth muscle cells, and platelets, where it performs critical biological functions.^[^ ^47^ ^]^ Similarly, HSPA5 has been investigated as a potential therapeutic target on the cell surface of some leukemias and solid tumors.^[^ ^48^, ^56^, ^57^ ^]^ Preclinical development of chemical or antibody drug candidates targeting HSP90B1 is underway across multiple cancer types, such as breast and colorectal cancers.^[^ ^55^, ^58^, ^59^, ^60^ ^]^ In the present study, we identified additional AML‐associated neoantigens, including csHSP90B1, which may be a promising candidate due to its known role as a conserved cancer therapeutic target. Primary patient samples often consist of mixed populations of normal and leukemic cells, which might be deemed as the observed variability in neoantigen‐positive subpopulations. Consistent with the phenomenon of malignant subpopulation variability observed in patient samples supported by studies on other potential neoantigens in AML, such as CD123, CLL‐1, and CD33,^[^ ^30^, ^61^ ^]^ by performing flow cytometry assay, our data show that the tested samples from AML patients, including all the four FLT3‐ITD^+^ cases, contained a distinct subpopulation with prominent csHSP90B1 signals, which were undetectable in samples from healthy donors (Figure 3k,l). Moreover, nearly no csHSP90B1 signals were detected on the surfaces of healthy PBMCs or bone marrow (BM) cells, including CD3^+^ T cells, CD19^+^ B cells, CD56^+^ NK cells, and CD34^+^ HSPCs (Figure 3m,n; Figure S8a–d, Supporting Information). This phenomenon exhibits particular prominence within a specific subset of AML, notably the FLT3‐ITD^+^ cases, and the working model is depicted in Figure 3o. While comprehensive profiling of neoantigen expression (particularly cell‐surface chaperone mis‐localization) across diverse healthy human tissues is impractical, we have performed flow cytometry experiments using murine tissue/organ and analyses revealed no detectable cell‐surface neoantigen signals (Figure S9a,b, Supporting Information), supported by the high molecular conservation. Collectively, these results underscore the role of these neoantigens, including csHSP90B1, as novel AML‐associated targets with potential for immunotherapeutic interventions.

**Figure 4:**
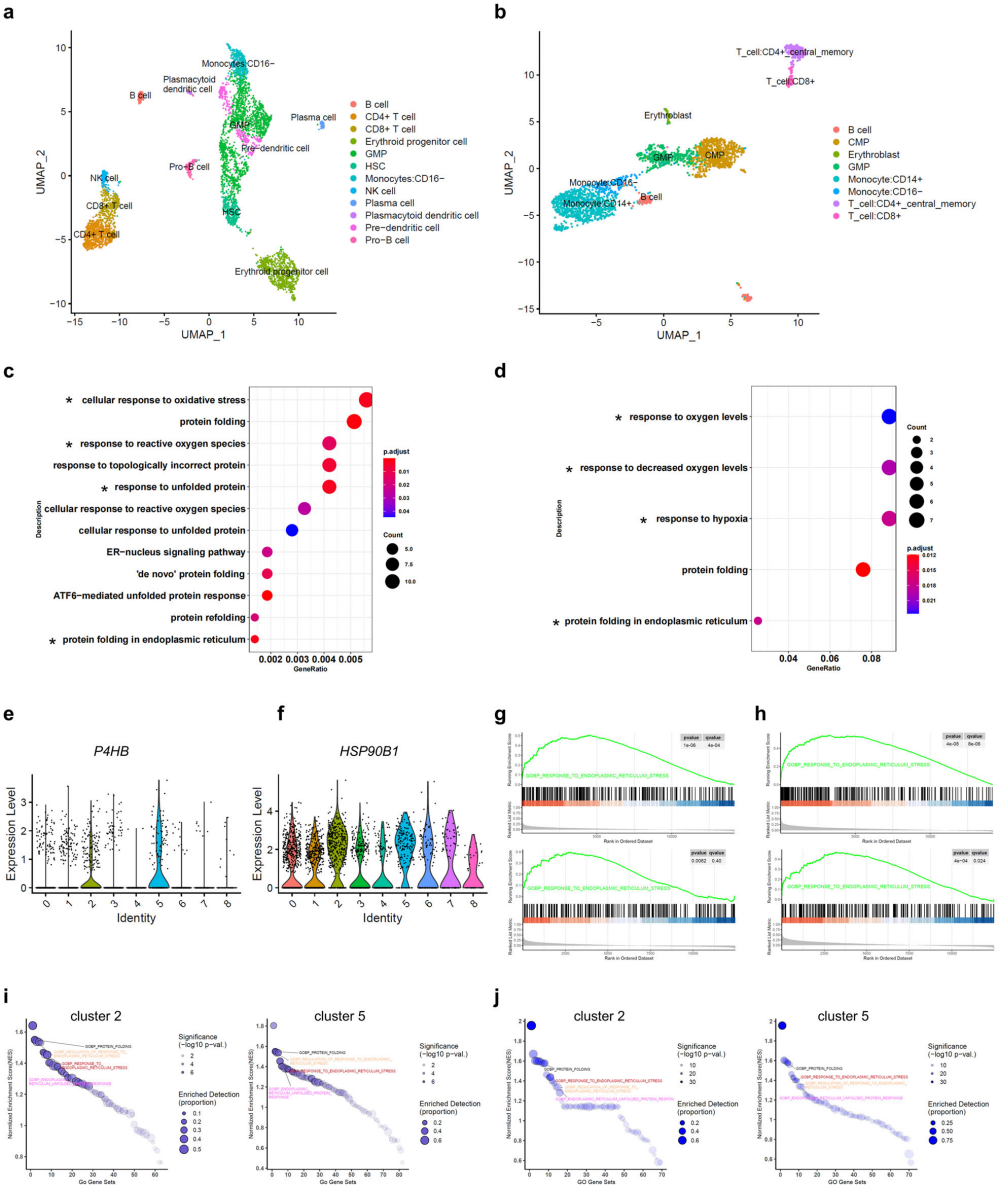
Single‐cell transcriptome reveals putative malignant cells endure chronic oncogenic stress, inducing chaperone re‐localization, as therapeutic targets. a) Uniform Manifold Approximation and Projection (UMAP) visualization of integrated datasets from bone marrow (BM) samples of four healthy donors. b) UMAP visualization of integrated datasets from the BM samples of four FLT3‐ITD^+^ AML patients. c,d) Gene ontology (GO) enrichment analysis for genes with significantly upregulated expression in Clusters 2 and 5 (*avg_logFC > log_2_(1.5), p_val_adj < 0.05*). e,f) Violin plots displaying the expression of two selected genes across all clusters in FLT3‐ITD^+^ AML patients, with elevated expression levels observed in Clusters 2 and 5 compared with other clusters. g,h) Single gene GSEA functional enrichment analysis based on the gene expression profiles correlated with *P4HB* (g) or *HSP90B1* (h) in cluster 2 (up panel) and cluster 5 (down panel). i,j) GSEA plots of the main enriched GO functional gene sets of *P4HB* (i) or *HSP90B1* (j) with NES>0 for the cluster 2 (left panel) and cluster 5 (right panel) cells. Detection is based on the enriched genes for each GO term.

### 2.4 Single‐Cell Transcriptional Analysis Unravels the Intervention Rationale

Having seen that cultured AML cells endure chronic oncogenic ER stress, we next aimed to investigate the primary AML cells by single‐cell RNA sequencing (scRNA‐seq) transcriptional analysis using public datasets. To do so, we first analyzed four cases with normal BM cells and performed Uniform Manifold Approximation and Projection (UMAP) analysis.^[^ ^62^ ^]^ The UMAP plot revealed 14 cell clusters, identified as 12 subpopulations based on canonical marker genes (**Figure** **4a**; Figure S10a–d, Supporting Information). These included hematopoietic stem cells, granulocyte–monocyte progenitors, cells of lymphoid, erythroid, and myeloid lineages, and others. Next, we examined cellular hierarchies in AML by using scRNA‐seq data from four cases of FLT3‐ITD^+^ AML, also derived from public datasets.^[^ ^62^ ^]^ UMAP analysis identified 9 cell clusters corresponding to 8 subpopulations, characterized using canonical marker genes (Figure 4b; Figure S11a–c,g, Supporting Information). Notably, the most consistent phenotypes across patients were the enrichment of common myeloid progenitors, granulocyte–monocyte progenitors, and monocyte subpopulations (Figure S11d–f, Supporting Information). The cell clusters between normal individuals and FLT3‐ITD^+^ AML patients revealed overlapping subpopulations, such as granulocyte–monocyte progenitors, monocytes, erythroid cells, and lymphoid cells (Figure 4a,b). However, a volcano plot analysis of gene expression in specific subpopulations revealed significant differences between the two groups. Two clusters corresponding to the granulocyte–monocyte progenitor and *CD16*‐negative monocyte in FLT3‐ITD^+^ AML patients exhibited distinct transcriptional profiles compared with their counterparts in healthy individuals. These subpopulations showed significant enrichment in GO terms related to cellular response to oxidative stress, response to hypoxia, response to unfolded protein, protein folding in endoplasmic reticulum stress, and ATF6‐mediated unfolded protein response (Figure 4c,d). In addition, these stress‐associated subpopulations displayed marked overexpression of chaperone transcripts such as *P4HB* and *HSP90B1* (Figure 4e,f). Single‐gene Gene Set Enrichment Analysis (GSEA) and GSEA plots of the main enriched GO functional gene sets revealed a significantly positive normalized enrichment score (NES) for both chaperones, demonstrating that genes in the GO terms, including ER stress response, are positively correlated with target genes (*P4HB, HSP90B1*) in these cell subpopulations (Figure 4g–j). Significantly upregulated chaperones, including *HSP90B1* and *P4HB*, in identified myeloid cells rather than non‐myeloid cells from the bone marrow samples of FLT3‐ITD^+^ AML patients were observed, but not in healthy donors (Figure S12a–d, Supporting Information). Collectively, these results suggest that putative leukemic subpopulations endure oncogenic cell stress at the single‐cell level, offering potential targets for therapeutic intervention.

### 2.5 The csHSP90B1‐CAR‐NK Cells Selectively Kill AML Cells in the Culture

**Figure 5:**
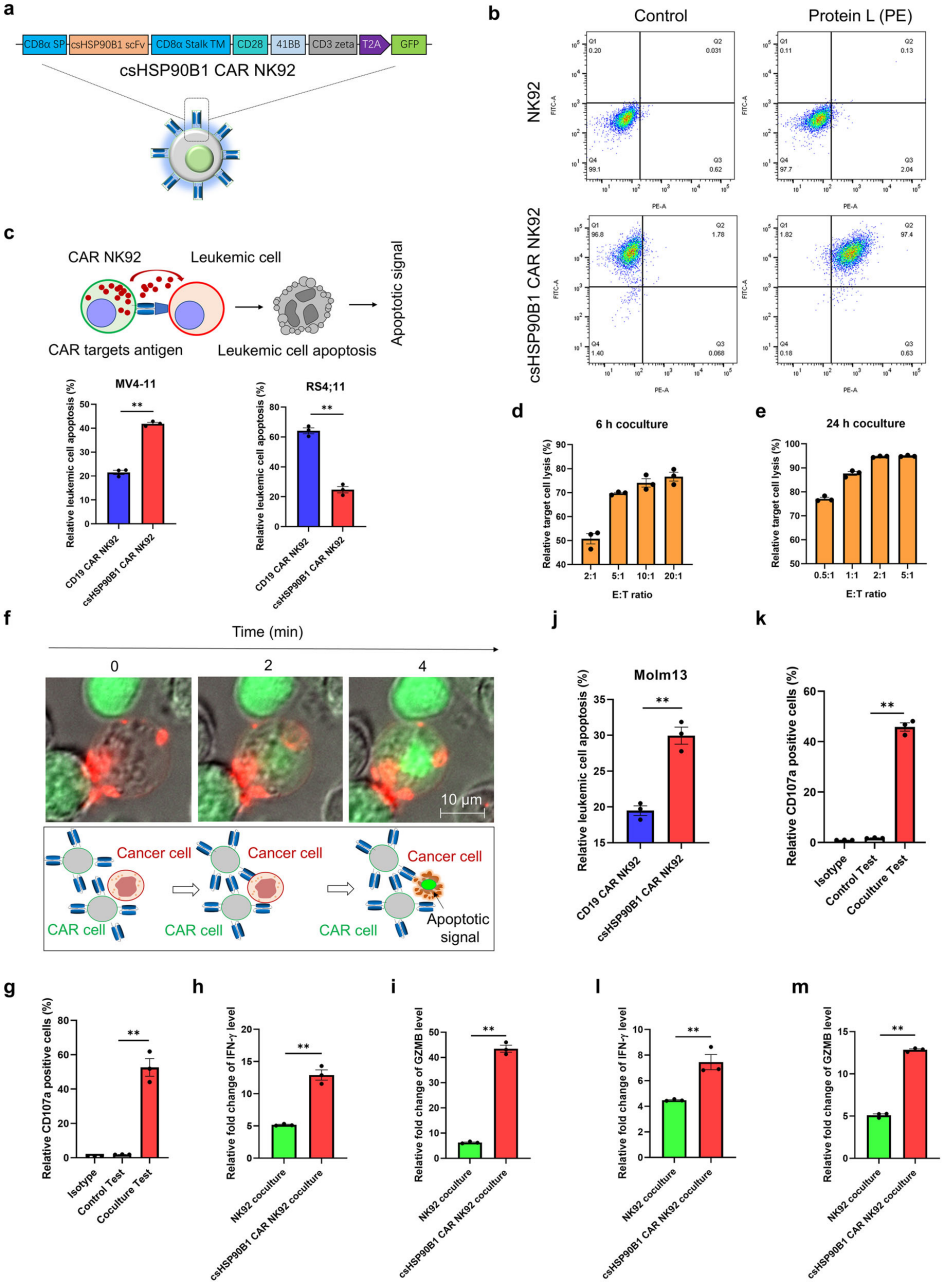
The csHSP90B1 CAR NK cells selectively kill AML cells by targeting the surface‐localized chaperone HSP90B1. a) Design schematic of the CAR structure in NK92 cells. b) Flow cytometry analysis of GFP and Protein L (PE) signals in stably transfected NK92 cells following sorting and enrichment. c) Detection of target cell apoptosis in the co‐culture system. Upper panel: Principle of apoptosis detection using flow cytometry. Lower panel: Relative apoptosis percentage of target cells (MV4‐11 or RS4;11) after 6 h of co‐culture with CAR NK92 cells at an E/T ratio of 2:1. Data represented as mean ± SEM, *p*‐value is calculated using Student's *t*‐test, *n* = 3, **p < 0.01. d,e) Nano‐luciferase‐expressing MV4‐11 cells co‐cultured with csHSP90B1 CAR NK92 cells at different E/T ratios for 6 and 24 h, respectively. Data represented as mean ± SEM, *n* = 3. f) Real‐time observation of the apoptosis process, showing red fluorescence‐labeled MV4‐11 cells being attacked by csHSP90B1 CAR NK92 cells. g) Relative percentage of cell surface CD107a‐positive cells in csHSP90B1 CAR NK92 cells, cultured alone or with MV4‐11 cells at an E/T ratio of 0.5:1 for 24 h. Data represented as mean ± SEM, *p*‐value is calculated using Student's *t*‐test, *n* = 3, **p < 0.01. h,i) Relative fold changes in interferon‐gamma (IFN‐γ) and granzyme B (GZMB) levels in the co‐culture supernatants of MV4‐11 cells paired with csHSP90B1 CAR NK92 cells, compared with co‐cultures with standard NK92 cells for 24 h at an E/T ratio of 0.5:1. Data represented as mean ± SEM, *p*‐value is calculated using Student's *t*‐test, *n* = 3, **p < 0.01. j) Relative apoptosis percentage of Molm13 cells after 6 h of co‐culture with csHSP90B1 CAR‐NK92 cells at an E/T ratio of 5:1. Data represented as mean ± SEM, *p*‐value is calculated using Student's *t*‐test, *n* = 3, **p < 0.01. k) Relative percentage of cell surface CD107a‐positive cells in csHSP90B1 CAR NK92 cells, cultured alone or with Molm13 cells at an E/T ratio of 0.5:1 for 24 h. Data represented as mean ± SEM, *p*‐value is calculated using Student's *t*‐test, *n* = 3, **p < 0.01. l,m) Relative fold changes in IFN‐γ and GZMB levels in the co‐culture supernatants of Molm13 cells paired with csHSP90B1 CAR NK92 cells, compared with co‐cultures with standard NK92 cells for 24 h at an E/T ratio of 0.5:1. Data represented as mean ± SEM, *p*‐value is calculated using Student's *t*‐test, *n* = 3, **p < 0.01.

The cell‐surface localization of chaperones in the AML cells noted in this study prompted speculation that these chaperones, akin to CD19 in ALL, might serve as therapeutic targets, particularly in FLT3‐ITD^+^ AML patients. HSP90B1 expression was higher in malignant cells,^[^ ^63^, ^64^ ^]^ suggesting its targeting could impede cell survival, and several HSP90B1‐targeted drug candidates have emerged.^[^ ^46^, ^63^ ^]^ To target csHSP90B1‐positive AML cells, in the present study, we engineered csHSP90B1‐specific CAR natural killer (NK) cells by using the NK92 cell line. The CAR construct (scFv‐CD28‐4‐1BB‐CD3ζ) was inserted into a lentiviral vector, alongside a T2A self‐cleaving peptide and a truncated green fluorescent protein (GFP) marker, for monitoring infection efficiency (**Figure** **5**a). To functionally validate extracellular exposure of translocated chaperone proteins, flow cytometry was performed on viable leukemic cells using scFv (single chain variable fragment) derived from a chimeric antigen receptor (CAR) construct. The scFv incubation assay revealed robust cell surface binding signals (Figure S13a,b, Supporting Information), indicating specific recognition of extracellular epitopes on HSP90B1 endoplasmic reticulum chaperones of these living cells. These results provide direct experimental evidence that CAR‐derived scFv engages surface‐translocated chaperone complexes, confirming their accessibility as membrane‐exposed targets. Stable CAR‐NK92 cells were enriched through repeated sorting rounds, and the surface expression of scFv was confirmed through phycoerythrin (PE)‐labeled Protein L binding with the co‐expressed GFP fluorescence signals (Figure 5b). To prevent CAR‐induced apoptosis (fratricide) of effector cells, we first verified the absence of csHSP90B1 expression on both NK92 cells and CAR‐transduced NK92 cells (Figure S13c, Supporting Information). Subsequently, we evaluated the cytotoxic function and antigen‐specificity of the csHSP90B1‐CAR NK92 cells by using two distinct cell lines as in vitro models: MV4‐11 (csHSP90B1‐positive, CD19‐negative) and RS4;11 (csHSP90B1‐negative, CD19‐positive). The effector CAR‐NK92 cells were co‐cultured with these target cells at an effector‐to‐target (E:T) ratio of 2:1 for 6 h to assess targeted cell death. Before initiating co‐culture experiments, we labeled the target cells with a nontoxic, red fluorescent dye, CM‐DiI. Cell apoptosis was monitored using Caspase‐3/7 green reagent and assessed through flow cytometry (Figure 5c). The results suggested that csHSP90B1‐CAR NK92 cells specifically induced apoptosis in MV4‐11 cells, whereas CD19‐CAR NK92 cells selectively targeted RS4;11 cells, which highlighted the antigen‐specificity of the csHSP90B1‐CAR NK92 cells. Further experiments demonstrated that the co‐incubation of csHSP90B1‐CAR NK92 cells with MV4‐11 cells under a 2:1 E:T ratio for 12 h resulted in markedly enhanced apoptosis of MV4‐11 cells compared with control NK92 cells (Figure S13d, Supporting Information). To examine cell cytotoxicity, luciferase‐expressing MV4‐11 cells were co‐cultured with csHSP90B1‐CAR NK92 cells in varying E:T ratios. The csHSP90B1‐CAR NK92 cells exhibited significant cytotoxicity across all tested ratios during a 6‐h co‐culture, culminating in near‐complete lysis of MV4‐11 cells after 24 h, even at the lowest E:T ratios (Figure 5d,e). These results showed the dose‐ and time‐dependent cytolytic activity of csHSP90B1‐CAR NK92 cells, demonstrating their robust therapeutic potential against csHSP90B1‐expressing AML cells.

Using live‐cell imaging, we observed that csHSP90B1‐CAR NK92 cells rapidly induced apoptosis in MV4‐11 target cells upon engagement. This swift induction of cell death was marked by the activation of Caspase‐3/7 in the cancer cells, occurring within minutes of interaction (Figure 5f; Movie S1, Supporting Information). In addition, csHSP90B1‐CAR NK92 cells displayed a significant upregulation of CD107a on their surface following co‐culture with MV4‐11 cells, indicating an enhanced degranulation process (Figure 5g). In cytokine secretion assays conducted using enzyme‐linked immunosorbent assay (ELISA), we found that CAR‐mediated cytotoxicity was accompanied by a substantial increase in interferon‐gamma (IFN‐γ) and Granzyme B (GZMB) secretion in the co‐culture supernatants of the MV4‐11 and csHSP90B1‐CAR NK92 cells. These levels were markedly higher than those observed in co‐cultures involving nonengineered NK92 cells (Figure 5h,i).

To further evaluate the efficacy of csHSP90B1‐CAR NK92 cells in a broader context, we extended our investigation to include additional AML cell lines, such as Molm13 and THP‐1 (Figure 5j; Figure S13e–g, Supporting Information). Consistent with the above observations, csHSP90B1‐CAR NK92 cells effectively induced apoptosis in these csHSP90B1^+^ target cells. In addition, akin to FLT3 mutations, we further observed SKNO‐1 and Kasumi‐1 AML cell lines, both harboring c‐Kit mutations, also represented consistent csHSP90B1 expressions (Figure S13h,i, Supporting Information). Next, using the FLT3‐ITD^+^ AML cell line Molm13, we further assessed their functional activity by measuring CD107a expression through flow cytometry and the levels of cytokines, including IFN‐γ and Granzyme B, through ELISA. The results demonstrated that Molm13 cells markedly stimulated csHSP90B1‐CAR NK92 cells, enhancing their anticancer functionality, as evidenced by the increased levels of CD107a expression and elevated secretion of IFN‐γ and Granzyme B (Figure 5k–m). Collectively, these results in cultured cells suggested the robust immunological response elicited by csHSP90B1‐CAR NK92 cells.

**Figure 6:**
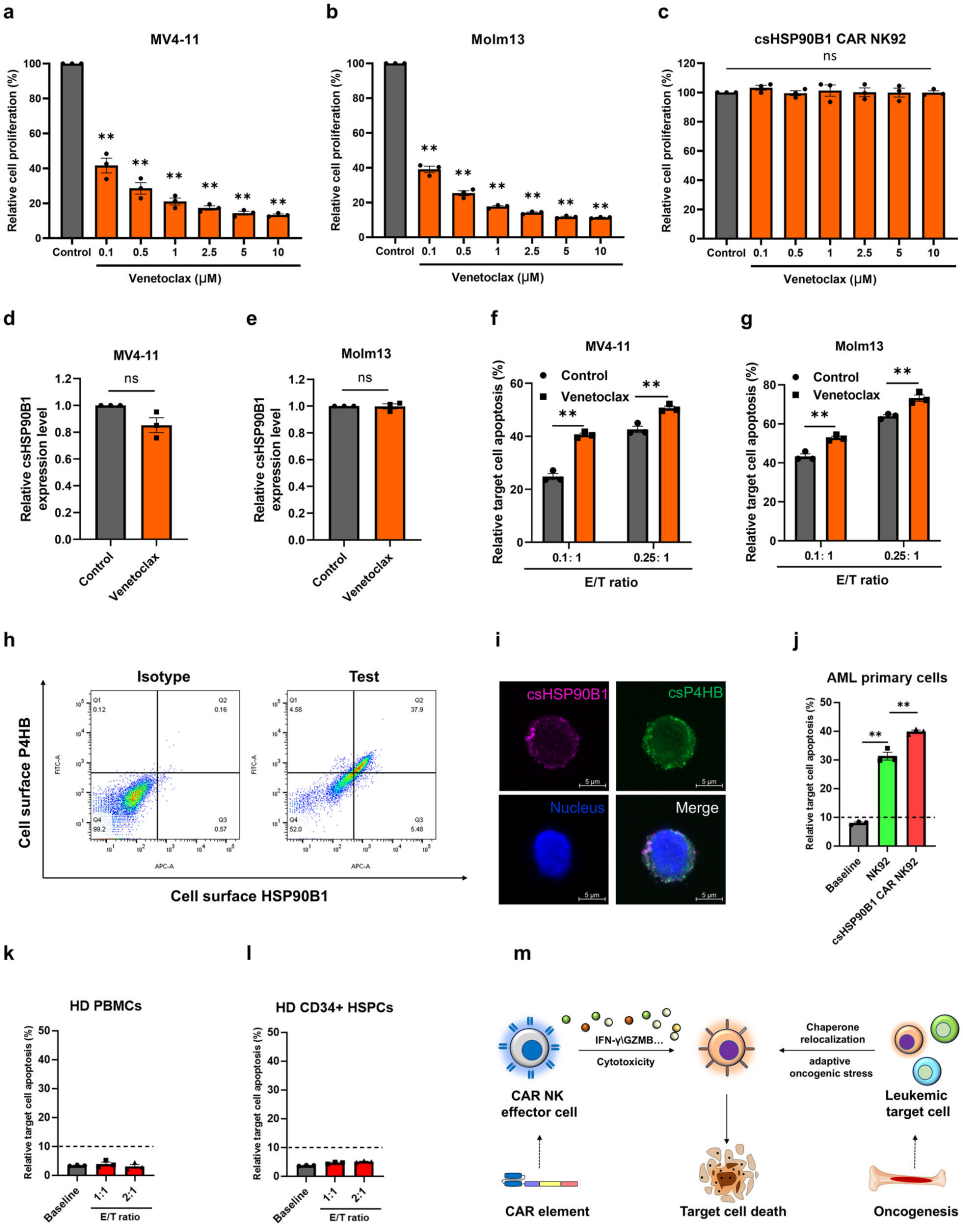
The csHSP90B1 CAR NK cells yield a more preferable outcome, combined with venetoclax, and selectively kill csHSP90B1^+^ AML primary cells but not healthy cells. a–c) Cell proliferation of MV4‐11 (a), Molm13 (b), and csHSP90B1 CAR NK92 cells (c) treated for 24 h with venetoclax at different concentrations. The data were normalized by the control group. Data represented as mean ± SEM, *p*‐values are calculated using Student's *t*‐test, *n* = 3, **p < 0.01, ns, not significant. d,e) The expression of csHSP90B1 in MV4‐11 (d) and Molm13 (e) after venetoclax treatment (10 nm) for 24 h. Data represented as mean ± SEM, *p*‐values are calculated using Student's *t*‐test, *n* = 3, ns, not significant. f,g) The csHSP90B1 CAR‐NK cells combined with low doses of venetoclax (10 nm) for 24 h displayed the notable benefits against MV4‐11 cells (f) and Molm13 cells (g). Data represented as mean ± SEM, *p*‐values are calculated using Student's *t*‐test, *n* = 3, **p < 0.01. h) Flow cytometry analysis of cell‐surface expression of P4HB and HSP90B1 in the non‐permeabilized primary cells from the FLT3‐ITD^+^ AML patient. i) Confocal microscopy images showing cell‐surface expression of P4HB and HSP90B1 in the non‐permeabilized FLT3‐ITD^+^ AML primary cells. j) Relative apoptosis percentages of primary cells from AML patients in co‐culture with parallel NK92 cells or csHSP90B1 CAR NK92 cells for 6 h at 1:1 E/T ratio, respectively. Data represented as mean ± SEM, *p*‐values are calculated using Student's *t*‐test, *n* = 3, **p < 0.01. k,l) Relative apoptosis percentage of healthy donor (HD) derived peripheral blood mononuclear cells (PBMCs, k) or CD34^+^ hematopoietic stem/progenitor cells (CD34+ HSPCs, l) in co‐culture with csHSP90B1 CAR NK92 cells for 6 h at different E/T ratios. Data represented as mean ± SEM, *n* = 3. m) Conceptual framework of how chaperone‐targeted CAR‐NK cells selectively attack malignant cells enduring chronic oncogenic stress.

It is widely acknowledged that BCL‐2 inhibitors, exemplified by the BCL‐2‐selective inhibitor venetoclax, hold substantial potential for AML treatment.^[^ ^65^ ^]^ Our cell proliferation assays demonstrated that AML cells (MV4‐11 and Molm13) manifested sensitivity to venetoclax treatment, whereas csHSP90B1‐CAR NK92 cells did not (**Figure** **6**a–c). Furthermore, we observed that venetoclax treatment exerted no pronounced effect on the expression of csHSP90B1 in target cells (Figure 6d,e). Notably, the combined treatment of csHSP90B1‐CAR NK92 cells with low‐dose venetoclax yielded a more preferable outcome when tested in MV4‐11 and Molm13 AML cells (Figure 6f,g). Collectively, these data imply the potential existence of combined therapeutic effects between csHSP90B1‐CAR NK92 cells and specific clinical drug therapies.

We next extended our analysis to evaluate the antitumor activity of csHSP90B1‐CAR NK92 cells on primary AML cells. A subset of primary cells from AML patients was found to express chaperones such as HSP90B1 and P4HB on their surface, as confirmed through flow cytometry and confocal microscopy (Figure 6h,i). Consistent with the findings from AML cell lines, flow cytometry analyses revealed that the treatment with csHSP90B1‐CAR NK92 cells induced potent cytotoxic effects on these primary AML cells (Figure 6j). Real‐time observations further supported these findings, showing the induction of apoptosis in csP4HB^+^ AML cells (labeled with an AF555‐conjugated antibody for P4HB recognition) within the co‐culture system with csHSP90B1‐CAR NK92 cells. Apoptosis was evidenced by the appearance of a green signal (Movie S2, Supporting Information). To assess the safety profile of csHSP90B1‐CAR NK92 cells, we examined their effects on normal PBMCs and CD34^+^ HSPCs from healthy donors. These analyses revealed that csHSP90B1‐CAR NK92 cells exhibited relatively minimal cytotoxicity against healthy cells (Figure 6k,l). As depicted in Figure 6m, the concept of csHSP90B1‐CAR NK92 cells selectively targeting and eliminating AML cells through CAR‐mediated interaction is validated, exhibiting significant anti‐AML activity and negligible cytotoxicity to normal cells.

### 2.6 Transcriptome Analysis of csHSP90B1‐CAR Cells Against Target Cancer Cells

**Figure 7:**
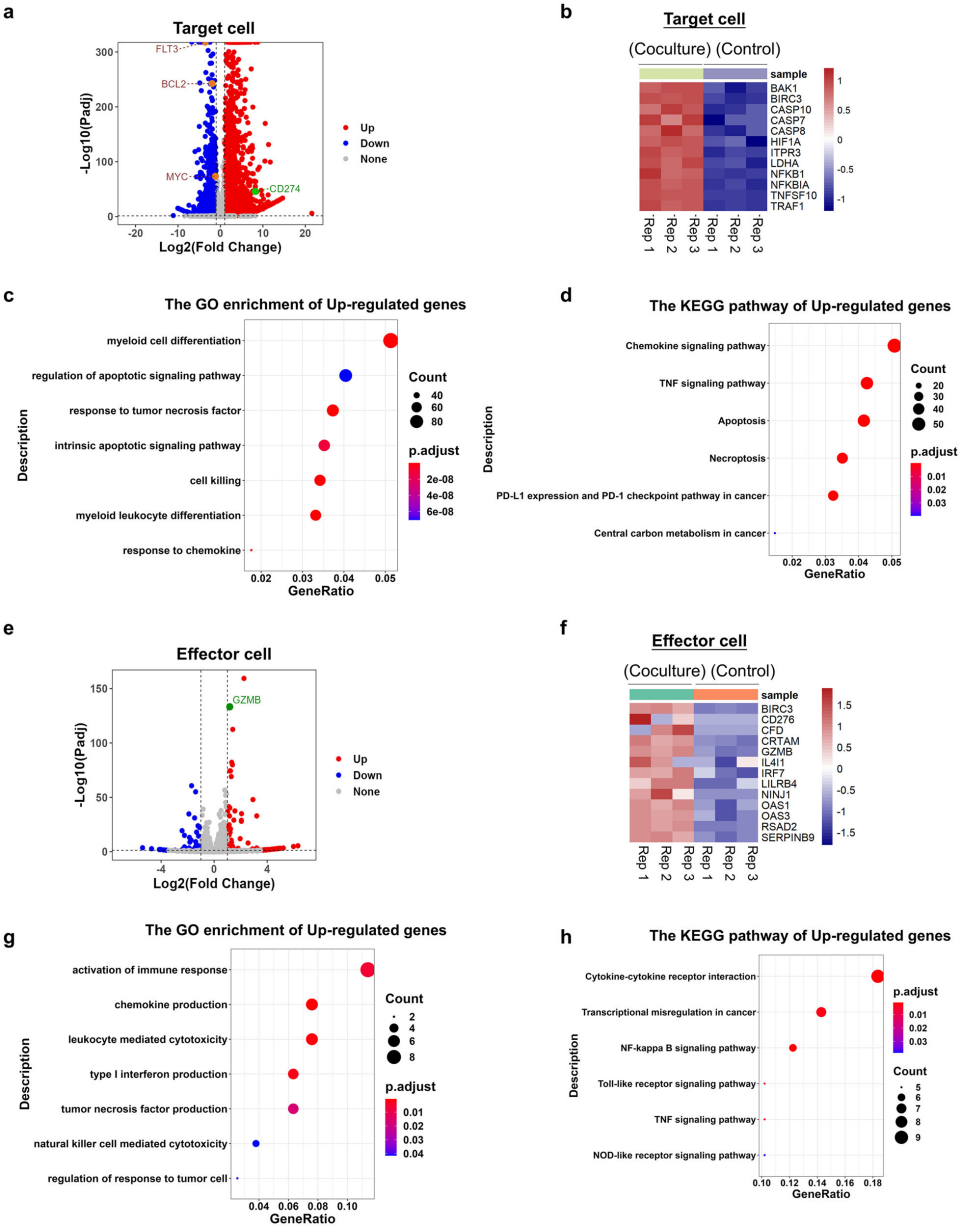
Transcriptome analysis of the csHSP90B1 CAR NK cells against target cancer cells. a) Volcano plot depicting the transcription profiles of MV4‐11 cells co‐cultured with csHSP90B1 CAR NK92 cells at an E/T ratio of 2:1 for 6 h, compared with non‐cocultured MV4‐11 cells. b) Heatmap illustrating the expression of selected genes associated with apoptosis and the PD‐L1/PD‐1 checkpoint pathway in MV4‐11 cells from (a). c) Gene ontology (GO) analysis of upregulated genes in MV4‐11 cells co‐cultured with csHSP90B1 CAR‐NK92 cells. d) Kyoto Encyclopedia of Genes and Genomes (KEGG) pathway analysis of upregulated genes in MV4‐11 cells co‐cultured with csHSP90B1 CAR‐NK92 cells. e) Volcano plot showing the transcription profiles of csHSP90B1 CAR‐NK92 cells co‐cultured with MV4‐11 cells at an E/T ratio of 2:1 for 6 h, compared with non‐cocultured csHSP90B1 CAR‐NK92 cells. f) Heatmap displaying the expression of genes related to immune response activation, NK cell‐mediated cytotoxicity, and regulation of responses to tumor cells in csHSP90B1 CAR NK92 cells from (e). g) GO analysis of upregulated genes in csHSP90B1 CAR NK92 cells co‐cultured with MV4‐11 cells. h) KEGG pathway analysis of upregulated genes in csHSP90B1 CAR NK92 cells co‐cultured with MV4‐11 cells.

To investigate molecular mechanisms underlying the effects of csHSP90B1‐CAR cells on target cancer cells, we performed RNA‐seq to analyze gene expression profiles in MV4‐11 target cells and csHSP90B1‐CAR NK92 effector cells. These analyses were conducted under both co‐culture conditions (E/T ratio of 2:1 for 6 h) and in isolation. RNA‐seq revealed significant changes in gene expression within co‐cultured MV4‐11 cells, with 2367 genes upregulated and 1880 genes downregulated compared with cells cultured independently (**Figure** **7**a,b). Notably, critical oncogenes, including *Myc* and *Flt3*, along with the anti‐apoptotic gene *Bcl2*, were significantly downregulated (Figure 7a). Moreover, transcriptomic analysis revealed upregulation of *CD274*, which encodes PD‐L1, in MV4‐11 cells following co‐culture with csHSP90B1‐CAR NK92 cells (Figure 7a). Although the role of PD‐L1 in AML has not been extensively studied compared with other cancers, it is recognized as a factor affecting disease prognosis. Increased secretion of IFN‐γ by csHSP90B1‐CAR NK92 cells was observed during co‐culture, which is known to induce PD‐L1 expression in cancer cells (Figure 5h,l; Figure S14a–d, Supporting Information). To confirm whether surface PD‐L1 expression is enhanced under co‐culture conditions, we measured surface PD‐L1 expression on MV4‐11 and Molm13 cells through flow cytometry. The results showed a significant upregulation of surface PD‐L1 on both cell lines following co‐culture with csHSP90B1‐CAR NK92 cells (Figure S14e–h, Supporting Information). These results suggested that the PD‐L1/PD‐1 axis might contribute to immune evasion in certain AML cases during disease progression and even during therapy progression.

Analysis of the effector cells revealed 83 upregulated and 58 downregulated genes in csHSP90B1‐CAR NK92 cells following co‐culture with target cells compared with their uncultured counterparts. Among these, *GZMB* was significantly upregulated, indicating its pivotal role in amplifying the cytotoxic capabilities of NK cells (Figure 7e,f). Comprehensive GO and Kyoto Encyclopedia of Genes and Genomes (KEGG) pathway analyses shed light on the co‐culture effects, aiding in the identification of pathways related to apoptosis induction in target cells and immune activation processes in effector cells (Figure 7c,d,g,h). These pathways included enhanced immune effector responses and mechanisms facilitating apoptosis, highlighting the interplay between target and effector cells during co‐culture. Taken together, these findings provide a detailed molecular understanding of cancer‐killing mechanisms mediated by csHSP90B1‐CAR NK92 cells. This map of interactions between target and effector cells offers valuable insights into the mechanistic basis of their therapeutic efficacy.

### 2.7 The csHSP90B1‐CAR‐NK Cells Exhibit Potent Anti‐AML Activity in Animal Models

**Figure 8:**
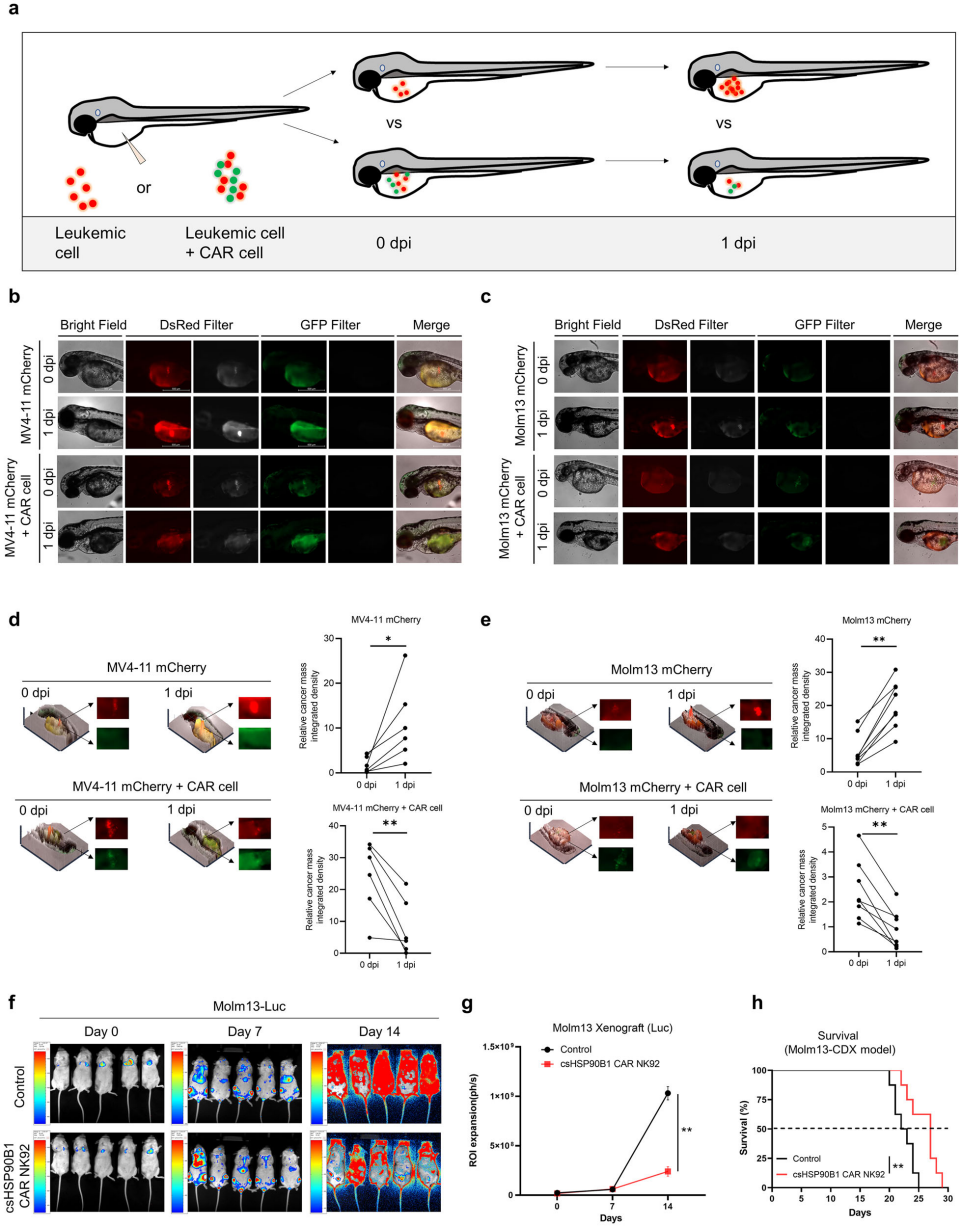
In vivo anti‐AML activity of csHSP90B1 CAR NK cells. a) Schematic for the anti‐cancer effects of csHSP90B1 CAR NK cells tested in a zebrafish xenograft model. b) Representative images showing MV4‐11‐mCherry cells alone or co‐injected with csHSP90B1 CAR NK92 cells in the zebrafish xenograft model. c) Representative images of Molm13‐mCherry cells alone or co‐injected with csHSP90B1 CAR NK92 cells in the zebrafish xenograft model. d) Left panel: Representative 2.5D display images of MV4‐11‐mCherry cells alone or co‐injected with csHSP90B1 CAR NK92 cells in the zebrafish model. Right panel: Quantification of relative fluorescence signals of MV4‐11‐mCherry cells in zebrafish xenografts without or with csHSP90B1 CAR NK92 cells. Data represented as separate values, *p*‐values are calculated using Student's *t*‐test, *n* = 6 in each group, *p < 0.05, **p < 0.01. e) Left panel: Representative 2.5D display images of Molm13‐mCherry cells alone or co‐injected with csHSP90B1 CAR NK92 cells in the zebrafish model. Right panel: Quantification of relative fluorescence signals of Molm13‐mCherry cells in zebrafish xenografts without or with csHSP90B1 CAR NK92 cells. Data represented as separate values, *p*‐values are calculated using Student's *t*‐test, *n* = 8 and 7 in each group, **p < 0.01. f) Representative images of bioluminescent Molm13‐luciferase cells in xenografted mice without or with csHSP90B1 CAR NK92 cell treatment. g) Bioluminescence quantification of Molm13‐luciferase cancer cells in the xenografted mice treated with or without csHSP90B1 CAR NK92 cells. Data represented as mean ± SEM, *p*‐value is calculated using Student's *t*‐test, *n* = 5 in each group, **p < 0.01. h) Survival curves of Molm13‐luciferase xenografted mice treated with or without csHSP90B1 CAR NK92 cells. The log‐rank test was applied to evaluate survival differences in mouse survival experiments. *n* = 8 in each group, **p < 0.01.

To evaluate the in vivo therapeutic potential of csHSP90B1‐CAR NK92 cells, we first used zebrafish embryos as an animal model and monitored their effect on target cancer cells (**Figure** **8**a). MV4‐11‐mCherry cells were injected into the yolk sacs of zebrafish embryos at 2‐day post‐fertilization (dpf), either alone or alongside csHSP90B1‐CAR NK92 cells with a 1:1 E/T ratio. At the start of the experiment (0 days post‐injection, dpi), mCherry fluorescence was clearly visible in the yolk sacs, confirming the presence of cancer cells. By 1 dpi, control zebrafish injected with MV4‐11‐mCherry cells alone displayed sustained and robust mCherry fluorescence, indicating continued viability of the cancer cells. By contrast, zebrafish co‐injected with csHSP90B1‐CAR NK92 cells showed a pronounced reduction in mCherry fluorescence (Figure 8b,d). Consistent results were obtained when Molm13‐mCherry cells were analyzed (Figure 8c,e). Collectively, our results confirmed the robust antitumor activity of csHSP90B1‐CAR NK92 cells, effectively reducing the population of target leukemic cells in a live organism.

To assess the safety and potential adverse effects of csHSP90B1‐CAR NK92 cells, we next conducted an experiment in NOD scid gamma (NSG) mice. The mice were administered IV injections of either 5 ×10^6^ csHSP90B1‐CAR NK92 cells, NK92 cells, or physiological saline as a control, twice a week for 2 weeks (Figure S15a, Supporting Information). The results indicated that treatment with csHSP90B1‐CAR NK92 cells did not cause obvious body weight loss when compared with NK92 cells or the saline control groups (Figure S15b, Supporting Information). After four rounds of injections, we still observed no substantial differences in body weight (Figure S15c, Supporting Information), and all mice survived, with no differences detected in survival rates up to one week after the final CAR cell injection (Figure S15d, Supporting Information). As a conserved member of the heat shock protein family, HSP90B1 exhibits high evolutionary conservation between mice and humans, with highly preserved amino acid sequence identity (Figure S16, Supporting Information). This functionally extends to its chaperone activity and client protein interactions, which are critical for their physiological and pathological functions. These findings suggested that csHSP90B1‐CAR NK92 cell treatment did not cause noticeable side effects or severe “on‐target off‐tumor” toxicities commonly observed with some CAR cell therapies. Consequently, preclinical safety profiles derived from murine models provide substantive guidance for anticipating human responses during the novel therapeutic form development. Next, we further evaluated the antitumor efficacy of csHSP90B1‐CAR NK92 cells in a therapeutic setting. The NSG mice were inoculated with Molm13 cells expressing luciferase on day 0, followed by the administration of effector cells or physiological saline controls. The csHSP90B1‐CAR NK92 cells demonstrated significant antitumor activity in vivo, as evidenced by a reduction in bioluminescence imaging signals from luciferase activity and a marked increase in overall survival compared with the saline control group in the cell derived xenograft (CDX) mice (Figure 8f–h). Taken together, these results collectively validated the therapeutic potential of using csHSP90B1‐CAR NK92 cells as a targeted approach for this specific AML subtype, while demonstrating their favorable safety and therapeutic profile in preclinical models.

## 3 Discussion

**Figure 9:**
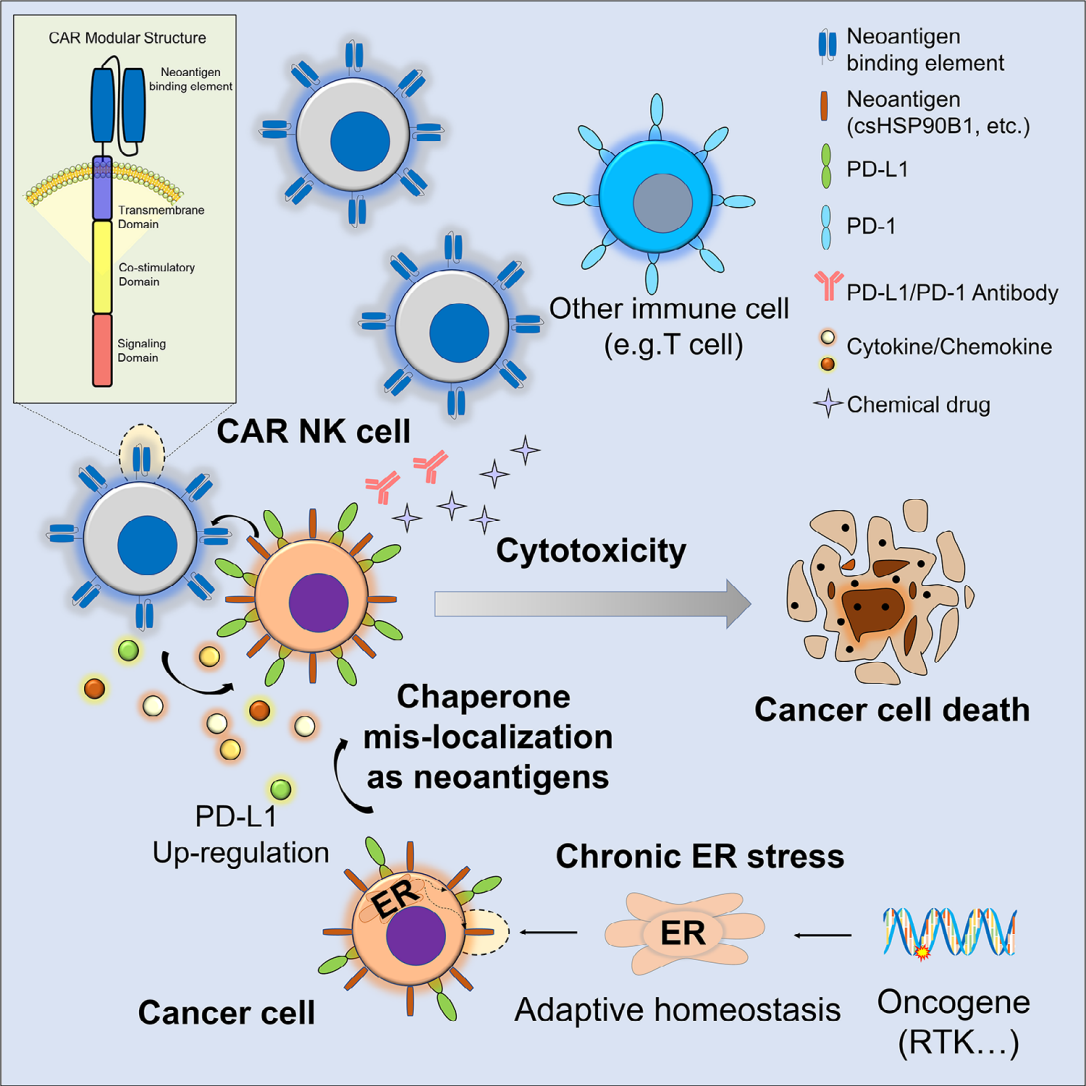
Proof‐of‐concept for a CAR cellular immunotherapy targeting cell‐surface chaperones triggered by ER stress in cancer treatment.

In this study, we identified potential neoantigens in AML by integrating comprehensive cell‐surface proteomics, transcriptome sequencing, and cell biology analyses. Notably, we discovered that certain ER chaperone proteins, typically confined to the ER, aberrantly localize to the cell surface under specific pathophysiological conditions. This mislocalization was strongly linked to chronic oncogenic stress in several AML subtypes, at least including the FLT3‐ITD^+^ AML subtype, positioning these chaperones as promising diagnostic markers and therapeutic targets. Additionally, single‐cell transcriptional analysis provided insights into the heterogeneous myeloid lineage landscapes of AML and revealed a strategic therapeutic rationale for targeting these cell‐surface chaperones. Based on the observations, as a proof‐of‐concept shown in **Figure** **9**, we engineered csHSP90B1‐CAR‐NK cells and demonstrated their capacity to selectively eliminate csHSP90B1^+^ AML cells both in vitro and in vivo using animal models. By exploiting the unique adaptive oncogenic stress profiles of malignant cells, this study lays the groundwork for developing innovative immunotherapeutic interventions in AML.

Given the extensive genomic variability observed in AML, therapeutic advancements continue to occur rapidly, with innovative strategies being developed to address the disease's heterogeneity.^[^ ^1^, ^12^ ^]^ Among the genetic mutations commonly observed in AML, FLT3‐ITD^+^ mutation is particularly noteworthy. This constitutively active form of FLT3 is present in ≈25–30% of AML patients and is associated with poor clinical outcomes.^[^ ^66^ ^]^ FLT3‐ITD^+^ AML patients were reported to have significantly higher relapse rates.^[^ ^67^ ^]^ Receptor tyrosine kinases (RTKs), such as FLT3, regulate cancer cell proliferation and survival.^[^ ^68^ ^]^ Targeted therapies using agents, such as midostaurin and quizartinib, both of which are RTK inhibitors, have demonstrated marked efficacy in improving overall survival rates in patients with FLT3 mutations.^[^ ^69^, ^70^, ^71^, ^72^ ^]^ Nevertheless, overcoming drug resistance remains a major challenge in the treatment of these patients. Notably, quizartinib treatment reduced but did not completely eliminate the expression of the targeted antigen in the remaining surviving leukemic cells in our study. This suggests that subsequent monotherapy with the developed CAR NK cells or combination therapy with other clinical agents, such as venetoclax, may still provide therapeutic benefits. This is supported by our preclinical data demonstrating that csHSP90B1 expression remains unaffected by venetoclax, and the combination of very low‐dose venetoclax with csHSP90B1 CAR NK cells enhances cytotoxicity against FLT3‐ITD⁺ AML cells. Recently, the retention of FLT3‐ITD^+^ proteins was found mainly in the ER and often induced proteotoxic stress and disrupted ER homeostasis, which might increase the sensitivity of cells to ER and oxidative stress, opening up new possibilities for identifying additional therapeutic approaches.^[^ ^13^, ^73^, ^74^, ^75^ ^]^ Interestingly, in this study, we found that specific ER chaperone proteins such as HSP90B1 abnormally translocate to the cell surface under particular pathophysiological circumstances, including FLT3‐ITD^+^ burdened AML.

CAR cell therapy has emerged as a revolutionary advance, serving as a novel form of immunotherapy.^[^ ^23^, ^26^, ^76^ ^]^ Despite successes, CAR‐T cell therapy faces challenges past neoantigen limits, chiefly high costs and toxicities, particularly cytokine release syndrome and immune effector cell‐associated neurotoxicity syndrome (ICANS).^[^ ^77^ ^]^ These adverse effects have spurred interest in alternative immune cell‐based therapies for cancer treatment in both clinical and preclinical settings.^[^ ^78^ ^]^ Among these, NK cell‐based immunotherapy has emerged as a promising strategy, offering novel avenues for combating a variety of cancers.^[^ ^79^ ^]^ NK‐92 cells have been extensively validated in preclinical and clinical settings for over three decades, offering distinct advantages as an “off‐the‐shelf” therapeutic platform. Unlike primary NK cells, which face challenges in isolation, expansion, and donor variability, NK‐92 cells provide a standardized and scalable alternative.^[^ ^80^ ^]^ Notably, over 100 cancer patients treated with unmodified or engineered NK‐92 cells (including CAR‐NK92) have shown clinical responses, even in refractory cases.^[^ ^43^, ^81^ ^]^ Given this established translational potential, we selected NK‐92 cells as a robust proof‐of‐concept model for the preclinical CAR‐NK development. Phase I/II clinical trials have demonstrated that pre‐irradiated CAR‐NK92 cells exhibit a significantly lower risk of severe toxicities, such as cytokine storms and neurotoxicity.^[^ ^80^, ^82^ ^]^ Additionally, CAR‐NK cells offer unique advantages, including the potent anticancer function and potential for universal, “off‐the‐shelf” cell therapy products.^[^ ^80^, ^83^, ^84^ ^]^ Unlike CAR‐T cells, CAR‐NK cells do not require patient‐specific customization, a feature made increasingly feasible with the advancements of cell manipulation techniques,^[^ ^43^, ^85^, ^86^, ^87^ ^]^ which are highly likely to lead to groundbreaking advancements in cancer treatment in the future.

HSP90B1, an ER stress protein, shares several characteristics with other members of the HSP90 protein family.^[^ ^17^ ^]^ HSP90B1 exhibits overexpression in various cancers, including lung, breast, myeloma, and other solid and hematologic malignancies.^[^ ^46^, ^63^ ^]^ This overexpression is significantly associated with the clinical stage of the disease and patient prognosis, and HSP90B1 was identified in pan‐cancer hallmarks to aid the development of a potential therapeutic target.^[^ ^63^ ^]^ As a conserved member of the heat shock protein family, its expression exhibits a negative association with CD8⁺ T cell infiltration while showing positive correlations with tumor mutational burden (TMB) and microsatellite instability (MSI). Furthermore, HSP90B1 expression is positively linked to tumor metabolism, cell cycle‐related pathways, and immune checkpoint gene expression, but negatively correlated with immunostimulatory genes. The HSP90B1 inhibitor significantly suppresses the proliferation of leukemic and solid tumor cells and downregulates PD‐L1 expression on cancer cell surfaces. Collectively, the expression patterns of HSP90B1 in diverse cancers and its associations with key biological processes, including tumor metabolism, cell cycle regulation, and immune checkpoint modulation, highlight its potential as a therapeutic target. Deeper mechanistic insights into HSP90B1's role in oncogenesis may inform novel anticancer strategies, providing a rationale for developing precision therapies targeting this chaperone protein. The use of autologous cancer‐derived HSP90B1 peptide complexes in vaccination has demonstrated promise in inducing cancer‐specific immune responses in several clinical trials.^[^ ^88^, ^89^, ^90^, ^91^, ^92^ ^]^ HSP90B1‐specific inhibitors such as PU‐WS13 have been developed to disrupt RTK activity, effectively impairing cancer cell survival and proliferation.^[^ ^58^, ^93^ ^]^ Notably, this present study provided evidence that under certain oncogenic stress, HSP90B1 could translocate to the cell surface, and demonstrated csHSP90B1‐CAR cell capacity to selectively eliminate malignant cells as a proof‐of‐concept. Consistent with prior studies,^[^ ^48^, ^51^, ^52^, ^56^, ^58^, ^94^, ^95^ ^]^ aberrant cell‐surface localization of chaperones (e.g., GRP78/Bip, HSP90B1) is observed across multiple malignant cell types, closely linked to oncogenic ER stress as a potential common hallmark, which might be closely associated with SRC activation. Divergent mechanisms underlying chaperone mislocalization in this context warrant further investigation. Combined with our findings on FLT3 or c‐Kit mutations in AML, aberrant tyrosine kinase signaling, including HER2 overexpression in breast cancer, represents a shared mutational hallmark across diverse cancer types. Notably, csHSP90B1's therapeutic potential in solid tumors is already well‐documented, underpinning preclinical/clinical development of small‐molecule inhibitors and monoclonal antibodies,^[^ ^58^, ^60^, ^96^, ^97^ ^]^ which substantiate the favorable safety profile of this target in these proof‐of‐concept studies. These results together emphasize the pivotal role of chaperones concerning aberrant expression and subcellular localization in cancer development and the tumor immune response, which warrants further investigation for targeted drug development. This study also has limitations that warrant further exploration. First, the zebrafish and immunodeficient murine models employed here inadequately recapitulate human immune‐tumor interactions. Future studies will utilize improved models such as iPSC‐derived AML/stromal co‐culture systems or humanized immune system mice to better mimic clinical pathophysiology and therapeutic response. Second, additional clinical samples will be collected to enhance validation of the neoantigen landscape in heterogeneous AML patients stratified by genetic subtypes and illustrate the correlating expression heterogeneity with therapeutic response to the AML precision treatment. Third, beyond hematological malignancies, the therapeutic potential of identified neoantigens should be assessed in relevant solid tumors. Additionally, engineered immune effector cells, including NK cells derived from cord blood, peripheral blood, or iPSCs, as well as T cells, could be explored to target these antigens in subsequent research. As for the future translational roadmap, to advance this present finding toward clinical implementation, comprehensive preclinical and translational studies remain essential. Key steps include rigorous evaluation of drug efficacy and safety profiles, detailed pharmacokinetic/pharmacodynamic (PK/PD) analyses, and development of scalable, cGMP‐compliant manufacturing processes. Subsequent clinical translation will also require phased human trials to establish safety, dosing regimens, and therapeutic efficacy, ultimately bridging laboratory discoveries to clinical practice.

In sum, our study presents a critical opportunity for neoantigen discovery to refine the classification of AML patients. Furthermore, it provides a strong rationale for employing chaperone‐targeted CAR cell therapies, such as csHSP90B1‐directed approaches, as part of a strategic framework for managing AML.

## 4 Experimental Section

### Cell lines and Primary Samples

The AML cell lines MV4‐11 (Research Resource Identifier, RRID:CVCL_0064), Molm13 (RRID:CVCL_2119), THP‐1 (RRID:CVCL_0006), SKNO‐1 (RRID:CVCL_2196) and Kasumi‐1 (RRID:CVCL_0589) along with the ALL cell line RS4;11 (RRID:CVCL_0093), the NK92 cell line (RRID:CVCL_2142), and HEK293‐T cells (RRID:CVCL_0063), were procured from Procell Life Science & Technology Co., Ltd (Wuhan, China) during the period between Jan, 2021 and Jun, 2022. BaF3 cells (RRID: CVCL_0161), obtained from iCell Life Science & Technology Co., Ltd. (Shanghai, China) in Jan 2023. All cell lines used in the experiments were confirmed to be free of contamination. Except NK92, all cells were cultured in RPMI 1640 medium (11875093, Gibco, CA, USA) supplemented with 10% heat‐inactivated fetal bovine serum and penicillin (100 U mL^−1^)/streptomycin (100 µg mL^−1^). NK92 cells were maintained in NK92 complete medium (TCH‐G292, Haixing Biosciences, Suzhou, China) with 200 U mL^−1^ human IL‐2. BaF3 cells were cultured in RPMI 1640 medium supplemented with mouse IL‐3 (HY‐P7062, MCE, Shanghai, China). Primary human cells isolated from patients and healthy donors at the First People's Hospital of Yunnan Province were cultured in complete medium (CM‐H030, Procell, Wuhan, China). All cell cultures were maintained in a humidified incubator at 37 °C with 5% CO_2_. The patients and healthy donors provided written informed consent to participate in this study. The study protocol was reviewed and received ethical approval (approval number KHLL2024‐KY129) from the Ethics Committee of the First People's Hospital of Yunnan Province.

### Antibodies and Reagents

The following reagents and antibodies were used in this study. Primary antibodies included anti‐endoplasmin/HSP90B1 (MABT196, Millipore), anti‐CD19 (18229, Abcam), Anti‐P4HB (ab2792, Abcam), anti‐PD‐L1 (ab205921, Abcam), anti‐GAPDH (181602, Abcam), Anti‐PDI (11245‐1‐AP, Proteintech), anti‐HSP90B1 (14700‐1‐AP, Proteintech), and anti‐CD107a (65051‐1‐Ig, Proteintech). Secondary antibodies included goat anti‐rabbit IgG H&L (Alexa Fluor 488; 150077, Abcam), goat anti‐mouse IgG H&L (Alexa Fluor 488; 150113, Abcam), goat anti‐rabbit IgG H&L (Alexa Fluor 555; 150078, Abcam), goat anti‐mouse IgG H&L (Alexa Fluor 555; 150118, Abcam), goat anti‐rabbit IgG H&L (Alexa Fluor 647; 150079, Abcam), goat anti‐mouse IgG H&L (Alexa Fluor 647; 150115, Abcam), goat anti‐rabbit IgG H&L (HRP; 205718, Abcam), and goat anti‐mouse IgG H&L (HRP; 97040, Abcam). Additional antibodies used included APC mouse anti‐human CD3 (UCHT1; 555335, BD Pharmingen), APC mouse anti‐human CD19 (HIB19; 555415, BD Pharmingen), APC mouse anti‐human CD56 (NCAM‐1, B159; 555518, BD Pharmingen), and APC mouse anti‐human CD34 (581; 555824, BD Pharmingen). Reagents used in this study included quizartinib (S1526, Selleckchem), venetoclax (HY‐1553, MCE), SRC inhibitor 1 (HY‐101053, MCE), IRE1α kinase‐IN‐2 (HY‐18509, MCE), PE‐conjugated protein L (RPL‐PP2H2, ACRO), CM‐DiI (C700, ThermoFisher), Fc block (564219, BD Biosciences), D‐Luciferin potassium (HY‐12591B, MCE), IL‐2 (HY‐P7037, MCE), Bright‐Glo Luciferase Assay Kit (E2620, Promega), CellTiter‐Glo Luminescent Cell Viability Assay Kit (G7571, Promega) and CellEvent Caspase‐3/7 Green Ready Probes (R37111, ThermoFisher). All reagents and antibodies were used as per the manufacturer's instructions.

### Cell‐Surface Biotinylation

Briefly, a cell suspension containing 5 × 10⁶ cells per sample was transferred into EP tubes and washed twice with DPBS at room temperature (RT). The cells were then resuspended in 100 µL of RT DPBS containing 40 µg HRP (Sigma) and 400 µm BxxP (APExBio) and incubated for 2 min at RT. To initiate labeling, 100 µL of RT DPBS containing 2 mm H_2_O_2_ was added to the suspension. The reaction proceeded for exactly 2 min at RT and was subsequently quenched by adding 200 µL of pre‐chilled DPBS containing 20 mm sodium azide, 20 mm sodium ascorbate, and 10 mm Trolox. Finally, the labeled cells were washed three times with DPBS for further processing.

### Affinity Capture of Biotinylated Proteins and on‐Bead Digestion

The biotin‐labeled cells were lysed on ice for 30 min by using RIPA lysis buffer (comprising 50 mm Tris‐HCl (pH 8.0), 150 mm NaCl, 0.2% SDS, 0.5% sodium deoxycholate, and 1% Triton X‐100) supplemented with 10 mm sodium azide, 10 mm sodium ascorbate, 5 mm Trolox, and 1 mm PMSF. The lysate was then sonicated and centrifuged at 16 000 × g for 15 min at 4 °C. Protein concentrations in the clarified supernatants were measured using the Pierce 660 nm Protein Assay Reagent. Biotinylated proteins were enriched from the lysates by using Pierce Streptavidin magnetic beads. The beads were prewashed twice with RIPA buffer before incubation with the lysates overnight at 4 °C. After incubation, the beads were sequentially washed twice with 1 mL RIPA buffer, once with 1 mL of 1 m KCl, once with 1 mL RIPA buffer, once with 1 mL of 2 m urea in 50 mm Tris‐HCl (pH 7.5), and twice again with 1 mL RIPA buffer. For MS preparation, the beads were further washed twice with 50 mm Tris‐HCl (pH 7.5) to remove detergent and twice with 25 mm ammonium bicarbonate (ABC). The beads were then resuspended in 200 µL of trypsin digestion buffer (25 mm ABC, 1 µg trypsin) per sample and digested on‐bead overnight at 37 °C with shaking at 1500 rpm on a thermomixer. The resulting digest was collected, followed by two washes with 3% formic acid (FA). The combined digests were acidified and desalted using C18 stage tips. The stage tips were conditioned sequentially with 50 µL of 100% methanol, 50 µL of 80% acetonitrile (MeCN) with 0.1% FA, and 50 µL of 0.1% FA (each step repeated twice). Acidified peptides were loaded onto the conditioned tips and washed twice with 50 µL of 0.1% FA. Elution was performed using 50 µL of 50% MeCN with 0.5% FA followed by 50 µL of 80% MeCN with 0.1% FA. The eluates were lyophilized and stored at −80 °C for subsequent liquid chromatography (LC)–MS analysis.

### LC‐MS Analysis

Desalted peptides were resuspended in 0.1% FA and separated using the EASY‐nLC 1200 System (Thermo Fisher Scientific). Peptides were analyzed on an Orbitrap Exploris 480 Mass Spectrometer (Thermo Fisher Scientific). Each sample was automatically injected onto a 75‐µm i.d. PepMap 100 (C18) nanocolumn at a flow rate of 5 µL min^−1^. The Orbitrap Exploris 480 was operated using a 120‐min gradient method. The gradient was programmed as follows: 9–35% mobile phase B over 95 min, 35–45% B over 13 min, 45–99% B over 2 min, followed by a 10‐min hold at 99% B. Mobile phase A consisted of 0.1% (v/v) FA in water, whereas mobile phase B contained 0.1% (v/v) FA and 80% (v/v) acetonitrile (ACN) in water. Data acquisition was performed in the data‐dependent mode. High‐energy collisional dissociation (HCD) MS/MS scans (resolution = 15 000) were acquired following MS1 scans (resolution = 60 000) in the 350–1500 m/z range. The normalized MS1 AGC target was set at 300%, and the MS2 AGC target was set at 75%. The HCD collision energy was fixed at 30%, with a dynamic exclusion time of 45 s.

### MS Data Processing

To compare cell‐surface proteins across different cell lines or PBMCs, two groups of raw data files were analyzed using MaxQuant v. 2.4.9.0. The enzyme trypsin/p was specified, with the maximum number of missed cleavages set to 4. The search database was the nonredundant human protein database (UP000005640_9606) obtained from UniProt. Both match‐between‐runs and label‐free quantification (LFQ) were enabled, whereas all other parameters were set to default. The LFQ data were further processed using Perseus software. After eliminating reverse hits and potential contaminants, proteins identified as having two or more unique peptides were retained for the subsequent analysis. Applying a log2(x) transformation, missing values were imputed from a normal distribution with a width of 0.3 and a downshift of 1.8 applied to the entire matrix. Welch's *t*‐test was used to assess statistical significance. To compare pulldown proteins between BxxP/H_2_O_2_‐treated and untreated samples, the raw data files were again processed using MaxQuant v. 2.4.9.0. The enzyme used was trypsin/p, and the maximum missed cleavage was set to 4. The database for the search was a nonredundant human protein database (UP000005640_9606) from UniProt. Match‐between‐runs and LFQ were used, whereas the other parameters were set to default. LFQ data were analyzed using Perseus software, with reverse hits and contaminants removed. Proteins identified by at least two unique peptides were retained, and the statistical significance was evaluated using Welch's *t*‐test. For each cell line or PBMC, a protein was considered significant if it was exclusively detected in labeled samples or exhibited a fold change of labeled/unlabeled ≥2 and a *p*‐value <0.05. Proteins classified as the cell surface/plasma membrane/extracellular proteins in the subcellular location of gene ontology (GO) annotation were identified as cell surface‐annotated proteins for subsequent analysis.

### Western Blotting

The cells were washed three times with PBS and lysed in cold lysis buffer. Protein electrophoresis was performed using 4–20% Bis‐Tris FastPAGE gels (TSINGKE) according to the manufacturer's protocol. Following electrophoresis, the proteins were transferred to nitrocellulose membranes, which were then blocked with 3% bovine serum albumin (BSA) in PBST (0.05% Tween‐20) for 1 h. For ordinary proteins, membranes were incubated with primary antibodies diluted in blocking buffer, followed by three 10‐min washes in PBST. Afterward, the membranes were incubated with HRP‐conjugated secondary antibodies diluted in blocking buffer for 1 h, followed by four 10‐min washes in PBST. For biotinylated proteins, membranes were incubated with HRP‐conjugated streptavidin (Thermo Fisher Scientific) diluted 1:10000 in blocking buffer for 1 h, followed by four 10‐min washes in PBST. Protein detection was performed using the Enhanced ECL Chemiluminescent Substrate Kit (YEASEN) and visualized using the Tanon‐5200Multi imaging system.

### Immune Fluorescence and Cell Imaging

The cells were fixed with 4% formaldehyde and blocked with Fc block and 0.5% BSA in PBS, without cell membrane permeabilization. The fixed cells were then incubated sequentially with primary antibodies, followed by fluorophore‐conjugated secondary antibodies. After thorough washing, the cells were mounted using the mounting medium (H‐1200, Vector Laboratories) and observed under a microscope (LSM 980, ZEISS) for analysis.

### Gene Knock‐Down (KD) Experiment

The shRNA lentivirus particles for HSP90B1 knockdown were produced by co‐transfecting pLKO.1‐shRNA plasmids into HEK293‐T cells with the packaging plasmids pMD2.G and psPAX. The shRNA sequence against human HSP90B1 is designed as follows: 5′‐CCT GTG GAT GAA TAC TGT ATT CTC GAG AAT ACA GTA TTC ATC CAC AGG TTT TTG‐3′.

### RNA Isolation and RT‐qPCR

Total RNAs were extracted by Super FastPure Cell RNA Isolation Kit (Vazyme) according to the manufacturer's instructions. Then, cDNA was synthesized by HiScript IV 1st Strand cDNA Synthesis Kit (Vazyme) using oligo (dT). The quantitative real‐time PCR reactions were performed by SupRealQ Purple Universal SYBR qPCR Master Mix (Vazyme) and LightCycler480 Real‐time PCR instrument (Roche). The relative mRNA expression was analyzed using the DDCt method. For each experimental sample, four technical replicates and three biological replicates were performed. The gene‐specific primers are listed as follows: *HSP90B1*‐F: 5′‐TAC CAA ACG GGC AAG GAC AT‐3′; *HSP90B1*‐R: 5′‐GAT ACC CTG ACC GAA GCG TT‐3′; *HSPA5*‐F: 5′‐TGG AAT GAC CCG TCT GTG C‐3′; *HSPA5*‐R: 5′‐TTT GGT TGC TTG GCG TTG G‐3′; *P4HB*‐F: 5′‐GAT TAC AAC GGG GAA CGC AC‐3′; *P4HB*‐R: 5′‐CGT CTT CCT CCA TGT CTG GC‐3′; *BAG3*‐F: 5′‐GTC ATC TGT CCA GGG TGC AT‐3′; *BAG3*‐R: 5′‐TTC TCG ATG GGT CAT GGG CT‐3′; *CAT*‐F: 5′‐ATG CAG GAC AAT CAG GGT GG‐3′; *CAT*‐R: 5′‐CGT TCA CAT AGA ATG CCC GC‐3′; *CYCS*‐F: 5′‐GAG CGG GAG TGT TCG TTG TG‐3′; *CYCS*‐R: 5′‐GCT TGC CTC CCT TTT CAA CG‐3′; *TUBB*‐F: 5′‐GGC GCT TAT CGA AGT GTG GT‐3′; *TUBB*‐R: 5′‐ACC CTT CCC CTA GAC ACT CG‐3′.

### Generation of CAR‐NK92 Cells

The CAR‐T2A‐GFP vector was constructed, and viral particles were produced by collecting the culture medium from the transfected HEK293‐T cells. NK92 cells were transduced with these viral particles, and CAR‐expressing NK92 cells were identified and sorted through flow cytometry based on GFP fluorescence. After multiple rounds of enrichment and sorting, stable CAR‐expressing NK92 cells were obtained. These cells were validated for GFP expression and CAR expression by the presence of double fluorescent signals: GFP signal and PE‐conjugated Protein L signal, following the manufacturer's instructions.

### Flow Cytometry Analysis and Cell Sorting

Target protein levels were analyzed through flow cytometry. Cells were prepared without the permeabilization step to detect cell‐surface proteins, respectively. Nonspecific staining was blocked using Fc block and 0.5% BSA in PBS. Primary antibodies, followed by fluorophore‐conjugated secondary antibodies, were applied for subsequent analysis using a FACSAria flow cytometer (BD Biosciences, USA) in accordance with the manufacturer's instructions. Cell sorting was performed based on relevant fluorescence signals.

### Cytotoxicity Experiment

Target cells were pre‐stained with the nontoxic red fluorescent dye CM‐DiI and co‐cultured with effector cells at specific E:T ratios and incubation times. For the apoptosis assay, apoptotic target cells were assessed using flow cytometry to detect green fluorescence signals generated by CellEvent Caspase‐3/7 Green Ready Probes. For the luciferase assay, target cells stably expressing (nano) luciferase were prepared. After co‐culture with effector cells, cell pellets were collected, and luciferase signals were measured to determine the remaining quantity of target cells. Detection was performed using the Bright‐Glo Luciferase Assay Kit. For the cell proliferation assay, cells were seeded in 96‐well plates for culture with or without the relevant chemical drug, and the relative cell viability tests were performed using the CellTiter‐Glo Luminescent Cell Viability Assay Kit. The SpectraMax iD5 microplate reader was used following the manufacturer's instructions.

### Degranulation Assay

Target cells were co‐cultured with effector cells under varying E:T ratios. The surface expression of CD107a on effector cells was assessed using the CD107a‐specific antibodies and analyzed through FACSAria flow cytometry (BD Biosciences, USA) following the manufacturer's instructions.

### Cytokine Release Assay

The production of IFN‐γ and GZMB was measured using ELISA kits (E‐EL‐H1617c, E‐EL‐H0108c, Elabscience) according to the manufacturer's instructions. Briefly, effector cells were seeded either alone or co‐cultured with target cells in 96‐well round‐bottom plates. After 24 h of incubation at an E:T ratio of 0.5:1, cytokine levels were quantified using a SpectraMax iD5 microplate reader following the provided protocols.

### RNA‐Seq Library Construction and Sequencing Experiment

Cells from different experimental groups were collected and washed three times with PBS. Total RNA was extracted using TRIzol reagent (15596026, Life Technologies) following the manufacturer's protocol. RNA‐seq libraries were prepared using 1–3 µg of total RNA and the VAHTS Universal V8 RNA‐seq Library Prep Kit for MGI (NRM605, Vazyme) in accordance with the manufacturer's instructions. The prepared libraries were sequenced on the MGISEQ‐2000 platform.

### Single‐Cell RNA Sequencing (scRNA‐Seq) Experimental Analysis

The datasets for the scRNA‐seq of AML BM cells and normal BM cells were retrieved from the Gene Expression Omnibus database under the accession number GSE116256. Based on clinical conditions, data from four FLT3‐ITD^+^ AML patients and four healthy donors were selected for analysis. The raw sequencing data, generated using Microwell‐seq, followed the experimental procedures and library construction steps. Post‐alignment, UMI counts for each sample underwent an initial cell filtration step. Cells with unique feature counts of 200–5000 and mitochondrial counts of less than 10% were retained for further analysis. Downstream analysis was conducted using the R package Seurat (v. 4.0.2). For normal BM cells, datasets were merged using the “merge” function and normalized with the “NormalizeData” function using the “LogNormalize” method. Principal component analysis was performed after scaling the data with the “ScaleData” function, using dimensions determined using the “PCElbowPlot” and “JackStrawPlot” functions. Data integration and batch correction were implemented using the “RunHarmony” function from the R package harmony (v. 0.1). Dimensional reduction was performed using the “RunUMAP” function in Seurat with the “harmony” reduction method, and clustering was executed using the “FindClusters” function with a resolution of 0.5. Signature genes for each cluster were identified using the “FindAllMarkers” function (parameters: only.pos = TRUE, min.pct = 0.25, logfc.threshold = 0.25). Only positive marker genes were considered potential biomarkers. Cell type annotation was initially performed using the R package SingleR (v. 1.4.1) with reference data from the Human Primary Cell Atlas via celldex (v. 1.0.0). Annotation inconsistencies were manually reviewed, and problematic clusters were re‐annotated using CellMarker2.0, based on published cell markers. The expression of cell‐determining markers was visualized using violin plots. An identical workflow was applied to the merged AML samples. Differentially expressed genes (DEGs) were identified after cell annotation. Positive DEGs with adjusted *p* values <0.05 and avg_log2FC >0.584 were used for GO enrichment analysis via clusterProfiler (v. 3.18.1). Additionally, clustering and annotation were conducted independently for three AML patients with sufficient cell counts.

### Zebrafish Xenograft Assay

The zebrafish (Danio rerio) model was utilized in compliance with the established guidelines for cancer xenograft assays. The study protocol was approved (approval number YNU20220193) by the Laboratory Animal Welfare Ethics Committee of Yunnan University. Briefly, the cells stably expressing fluorescent proteins were injected into the yolk sacs of zebrafish embryos at 48 h post‐fertilization (hpf) using glass needles and a FemtoJet injection system (Eppendorf, Germany). Following injection, the zebrafish were maintained at 32 °C, an increase from the standard 28 °C. Microscopic imaging was conducted at various time points to monitor fluorescence. The density of fluorescently labeled injected cells was analyzed using ImageJ software. At the conclusion of the experiments, all zebrafish were euthanized with an overdose of anesthesia to ensure humane treatment.

### Mouse In Vivo Experiment

Female NSG mice aged 6–8 weeks were used in this study, obtained from the Shanghai Model Organisms Center (Shanghai, China) and housed in the Laboratory Animal Center of Yunnan University. This study was conducted in compliance with Yunnan University's guidelines for the care and use of laboratory animals. All experimental procedures were reviewed and approved (approval number YNU20220193) by the Laboratory Animal Welfare Ethics Committee of Yunnan University. Cancer cell engraftment and therapeutic responses were assessed using bioluminescence (BLI) with the Photon Imager Optima System (Biospace Lab, France). Target and effector cells were injected intravenously (IV) as specified in the experimental protocols. For survival studies of cancer xenografted mice, 1 × 10⁷ effector cells per dose, twice a week for two weeks, were administered via IV injections supplemented with human IL‐2 to ensure immune cell activity.

### Statistical Analysis

All experiments were performed independently at least thrice. Statistical analyses were conducted using appropriate methods based on the experimental design using GraphPad Prism 8 software (GraphPad Software, San Diego, CA, USA). For comparisons between two groups, significance was assessed using Student's *t*‐test. For comparisons involving three or more groups, the analysis of variance was performed. The log‐rank test was applied to evaluate survival differences in mouse survival experiments. All data are presented as the mean ± standard error of the mean (SEM), and a *p*‐value of less than 0.05 was considered statistically significant.

### Data, Material, and Software Availability

The data supporting the conclusions of this article are included within the article or the supporting information. The mass spectrometry proteomics data generated in this study have been deposited to the ProteomeXchange Consortium via the iProX partner repository,^[^ ^98^, ^99^ ^]^ with the dataset identifier PXD058892. The raw sequencing data generated from the cell lines and primary cells in this study have been deposited in the NCBI's Gene Expression Omnibus and are accessible through GEO Series accession number GSE291245.

## Conflict of Interest

D.C., B.Z., and Y.L. have patent applications in the field of immunotherapy (ZL 2024 1 0547223.3; PCT/CN2025/071181). The remaining authors declare no competing interests.

## Author Contributions

Y.Z., Z.Z., and P.H. contributed equally to this work. D.C., Q.S., T.Y. and B.Z. conceived the project and designed the experiments. Y.Z. and W.W. conducted the MS experiments. Y.Z., W.W., Y.S., F.H., Y.L., Q.S., and B.Z. conducted the experiments. Y.Z., Z.Z., Y.L. B.Z. and D.C analyzed the data. P.H., Q.S., Y.Y., and N.Y collected samples and clinical information. Q.S., T.Y. B.Z. and D.C. wrote the manuscript. All the authors read and approved the final manuscript.

## Supporting information



Supporting Information

Supplemental Dataset 1

Supplemental Dataset 2

Supplemental Dataset 3

Supplemental Movie S1

Supplemental Movie S2

## Data Availability

The data supporting the conclusions of this article are included within the article or the supporting information. The mass spectrometry proteomics data generated in this study have been deposited to the ProteomeXchange Consortium via the iProX partner repository, with the dataset identifier PXD058892. The raw sequencing data generated from the cell lines and primary cells in this study have been deposited in the NCBI's Gene Expression Omnibus and are accessible through GEO Series accession number GSE291245.
