# Supplementary material for: Chronic ER Stress Triggers Cell‐Surface Chaperones as the Therapeutic Targets of CAR Cells in Acute Myeloid Leukemia
Source: Adv Sci (Weinh). 2025 Oct 23;13(5):e11573. doi: 10.1002/advs.202511573 (PMC12849903; doi:10.1002/advs.202511573)
Supplement: Supplementary file 1 — Supporting Information [file ADVS-13-e11573-s002.docx]

Supporting Information

Figure S1. Biotinylation of cell-surface proteins and analyses of cell-surface proteomic datasets. a) Immunofluorescence and cell imaging showing the biotinylation of cell-surface proteins. b) Western blot analysis confirming the biotinylation of cell-surface proteins. c) Number of proteins identified and analyzed through mass spectrometry. d-g) Volcano plots illustrating the differential expression of cell-surface proteins in various comparisons: MV4-11 versus RS4;11 (d), Molm13 versus RS4;11 (e), MV4-11 versus PBMC (f), and Molm13 versus PBMC (g). h) Top 30 gene ontology terms enriched for AML-specific cell-surface proteins identified as common hits across the four comparisons (d-g).


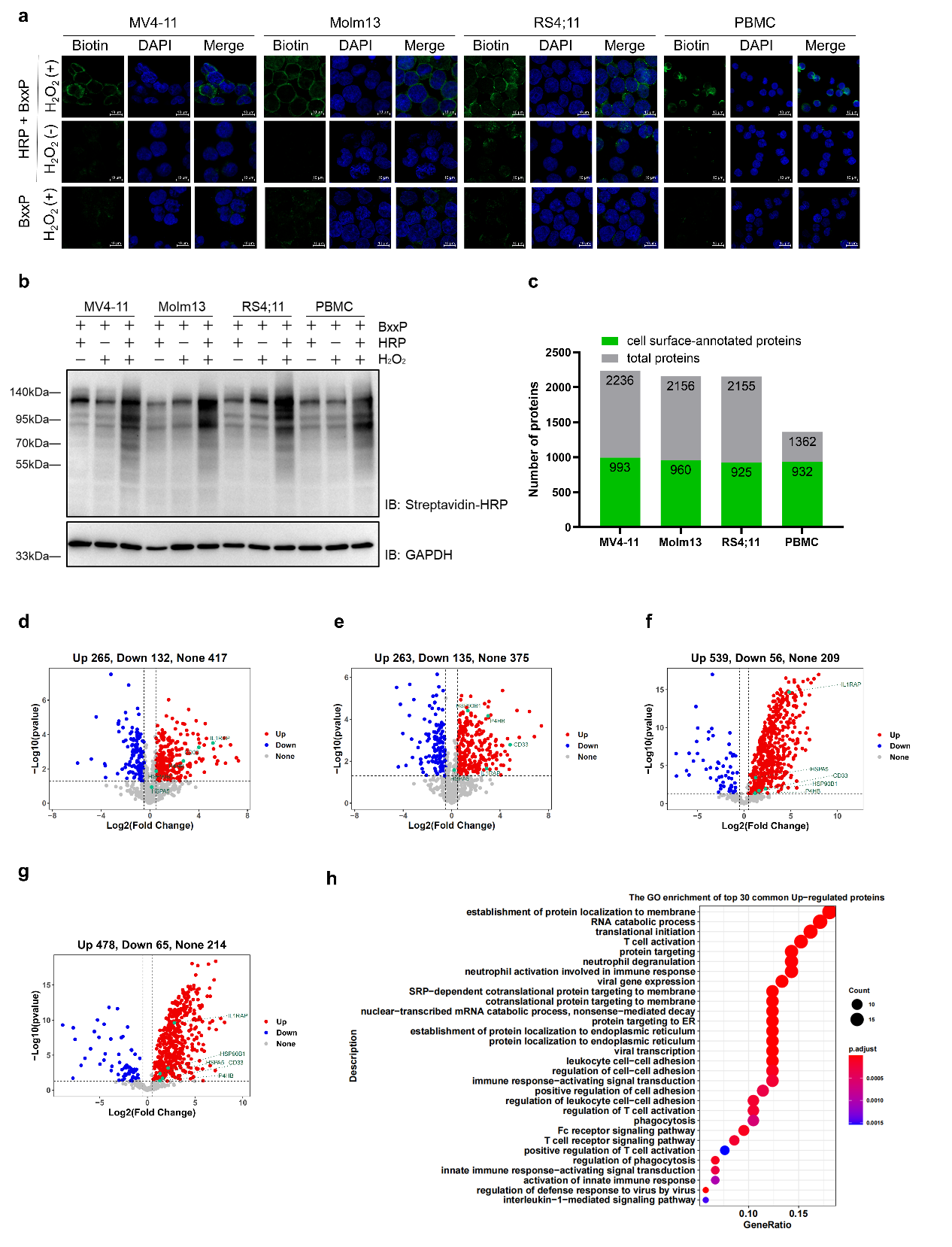


Figure S2. Transcription profiles in AML cells and the detection of potential cell-surface neoantigens in leukemic cells. a) Workflow illustrating the screening of ER stress-induced transcription profiles in HEK293-T cells. b) Volcano plots showing the transcription profiles in HEK293-T cells treated with thapsigargin (TG, 1 nM, 6 h). c) Gene ontology (GO) analysis of the upregulated genes in (b). d-g) Volcano plots comparing transcription profiles across different datasets: MV4-11 versus RS4;11 (d), Molm13 versus RS4;11 (e), MV4-11 versus PBMC (f), and Molm13 versus PBMC (g). h) Immunofluorescence images showing cell-surface expression of P4HB (csP4HB) in non-permeabilized MV4-11, Molm13, and RS4;11 cells. i) Flow cytometry analysis of csP4HB expression in living MV4-11, Molm13, and RS4;11 cells. j) Immunofluorescence images showing cell-surface CD19 (csCD19) expression in non-permeabilized MV4-11, Molm13, and RS4;11 cells. k) Flow cytometry analysis of csCD19 expression in living MV4-11, Molm13, and RS4;11 cells.


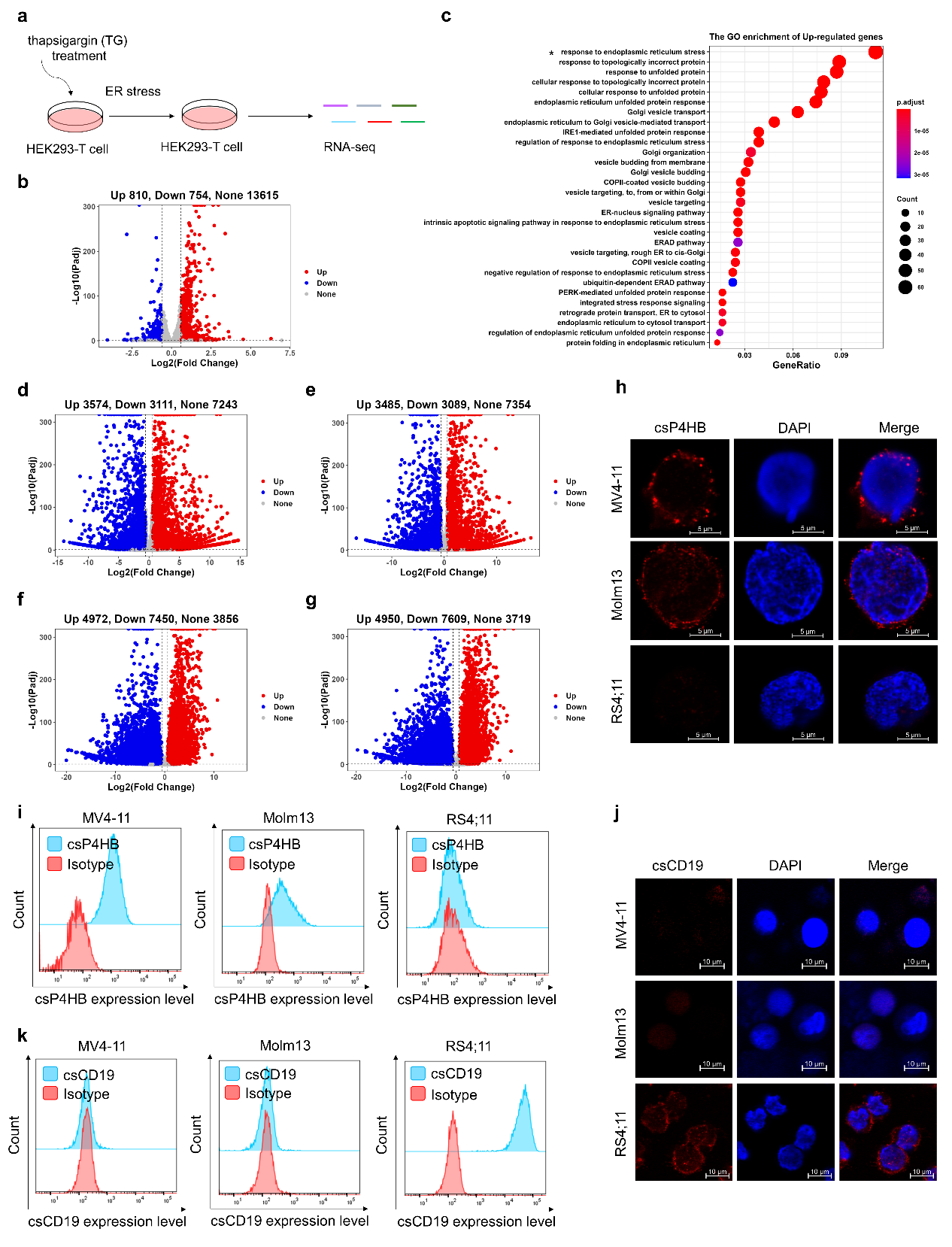


Figure S3. Validation of the key candidates by RT-qPCR for transcriptomic findings. a) RT-qPCR experiments for the key candidates in transcriptomic findings between MV4-11, Molm13 and RS4;11. b) RT-qPCR experiments for the key candidates in transcriptomic findings between MV4-11, Molm13 and healthy PBMC. c) RT-qPCR experimental validation for the key candidates regulated by quizartinib treatment in MV4-11 and Molm13 cells. Data represent as mean ± SEM, *p*-values are calculated using Student’s *t-*test, *n*=3, **p* < 0.05, ***p* < 0.01.


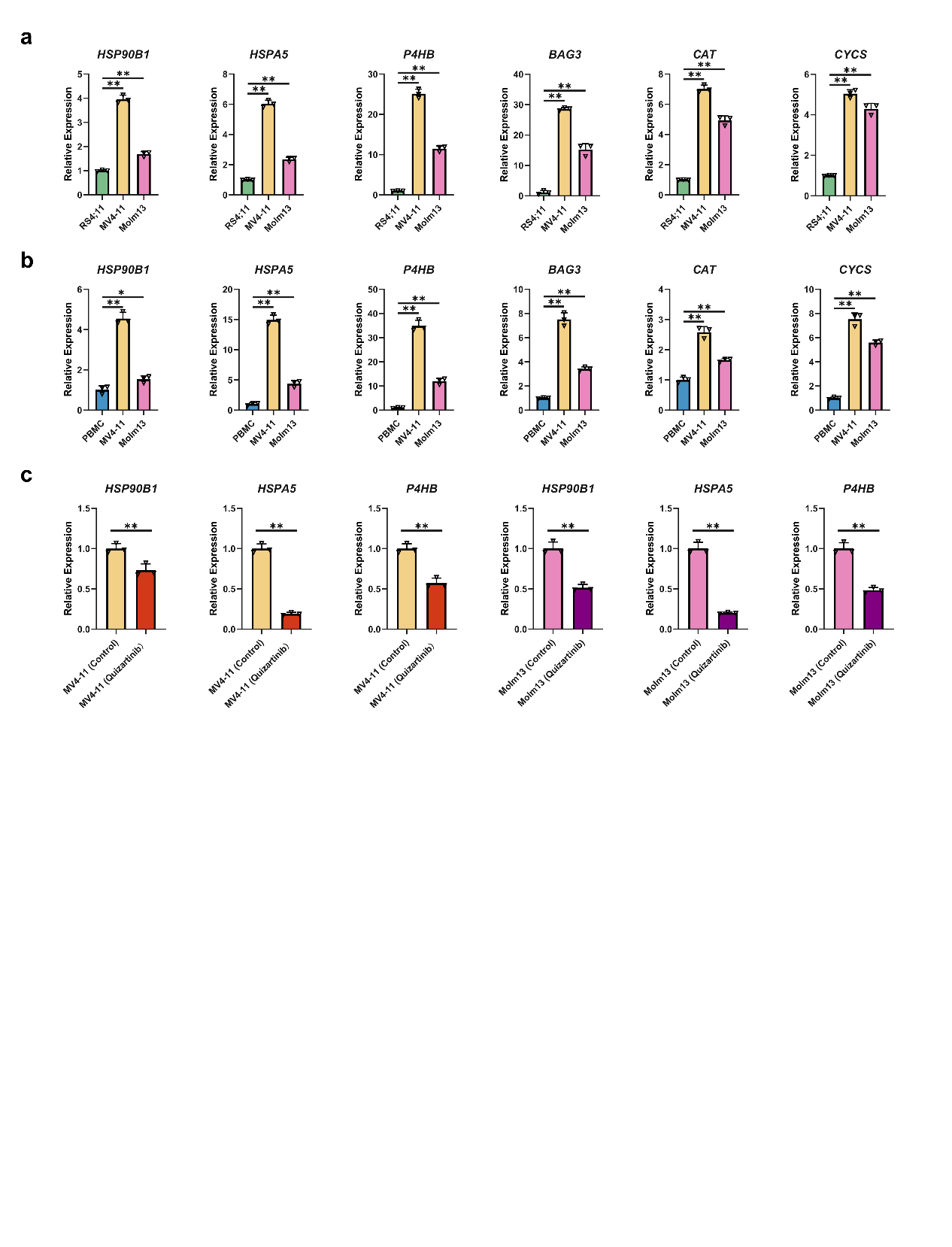


Figure S4. Validation of the antibody specificity and staining reliability for the HSP90B1 target. a) Flow cytometry analysis showing that shRNA-mediated HSP90B1 knockdown decreased csHSP90B1 expression on living MV4-11 cell surface. b) Quantification of csHSP90B1 expression levels in living MV4-11 cells from (a). Data represent as mean ± SEM of fluorescence intensity (MFI), *p*-values are calculated using Student’s *t-*test, *n*=3, ***p* < 0.01. c) RT-qPCR validation for the shRNA knockdown efficiency of HSP90B1 in MV4-11 cells. Data represent as mean ± SEM, *p*-values are calculated using Student’s *t-*test, *n*=4, ***p* < 0.01. d) Western blot analyses using this monoclonal antibody (employed in immunofluorescence studies) demonstrated minimal non-specific bands in whole cell lysates. The observed band correlated with HSP90B1, as confirmed by protein molecular weight markers. e) Overlapping signals from HSP90B1-GFP and fluorophore-conjugated antibodies targeting extracellular HSP90B1 were observed on the surface of non-permeabilized THP-1 leukemic cells that were engineered to overexpress the HSP90B1-GFP fusion protein.​ As a contrast, cell surface signals from fluorophore-conjugated antibodies targeting extracellular HSP90B1 were distinct and did not overlap with the signals from control GFP proteins in non-permeabilized THP-1 leukemic cells that were engineered to overexpress GFP.


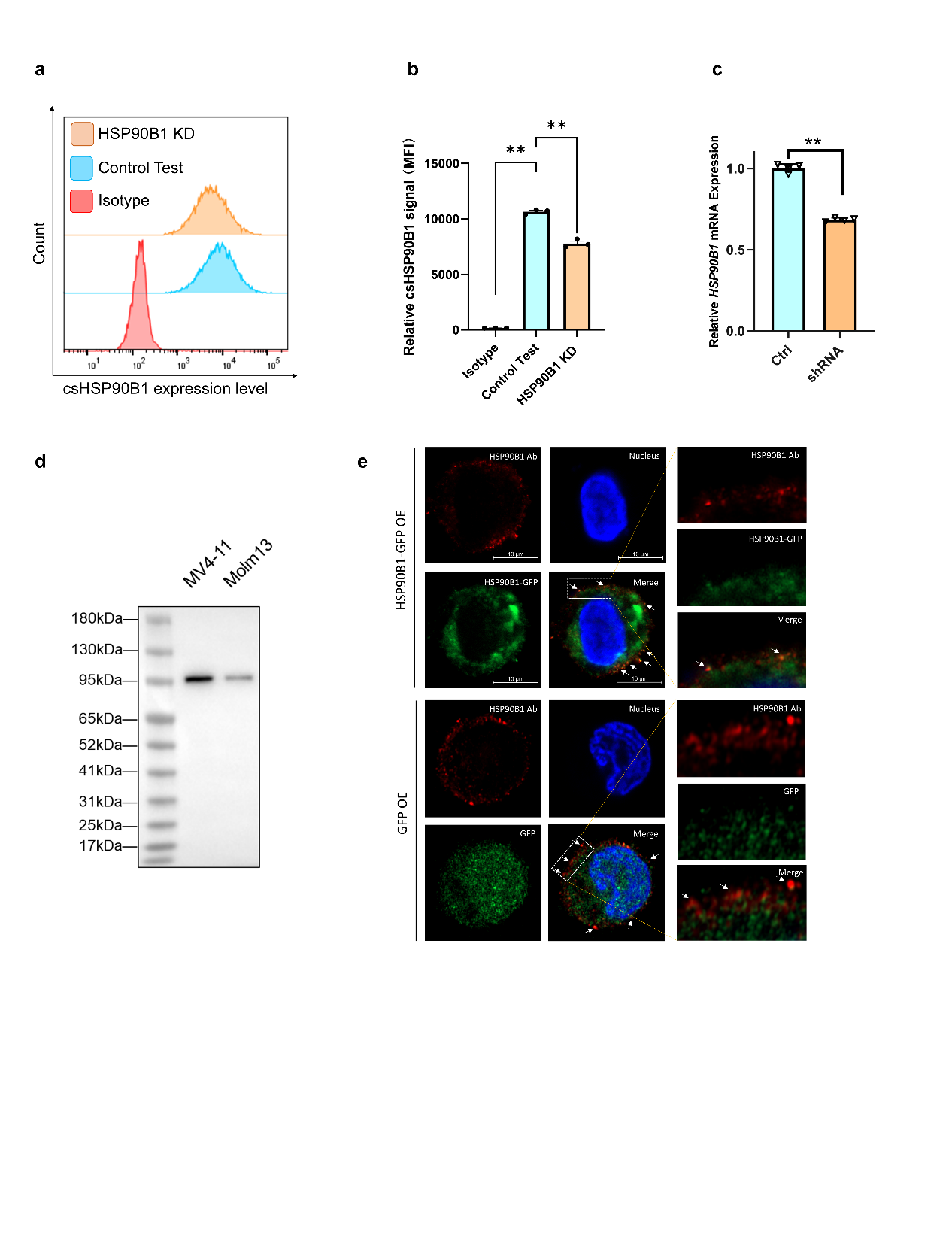


Figure S5. Transcription profiles and cell-surface neoantigens are closely associated with RTK. a) Volcano plot illustrating the transcription profiles in MV4-11 cells treated with quizartinib (100 nM, 8 h). b) Volcano plot illustrating the transcription profiles in Molm13 cells treated with quizartinib (100 nM, 8 h). c) Venn diagram showing commonly downregulated transcription profiles induced by quizartinib treatment in both MV4-11 and Molm13 cells. d, e) Quantification of csP4HB expression levels in living MV4-11 (d) and Molm13 (e) cells after quizartinib treatment (100 nM) at various time points, as analyzed through flow cytometry. Data represent as mean ± SEM of fluorescence intensity (MFI), *p*-values are calculated using Student’s *t-*test, *n*=3, **p* < 0.05, ***p* < 0.01. f, g) Flow cytometry analyses (f) and quantification (g) of csHSP90B1 expression levels in living BaF3 cells stably expressing *homo sapiens* (hs) wild-type (wt) FLT3 or hs FLT3-ITD^+^ proteins. Data represent as mean ± SEM of fluorescence intensity (MFI), *p*-values are calculated using Student’s *t-*test, *n*=3, ***p* < 0.01. h, i) Flow cytometry analyses (h) and quantification (i) of csP4HB expression levels in living BaF3 cells stably expressing hs wild-type (wt) FLT3 or hs FLT3-ITD^+^ proteins. Data represent as mean ± SEM of fluorescence intensity (MFI), *p*-values are calculated using Student’s *t-*test, *n*=3, ***p* < 0.01.


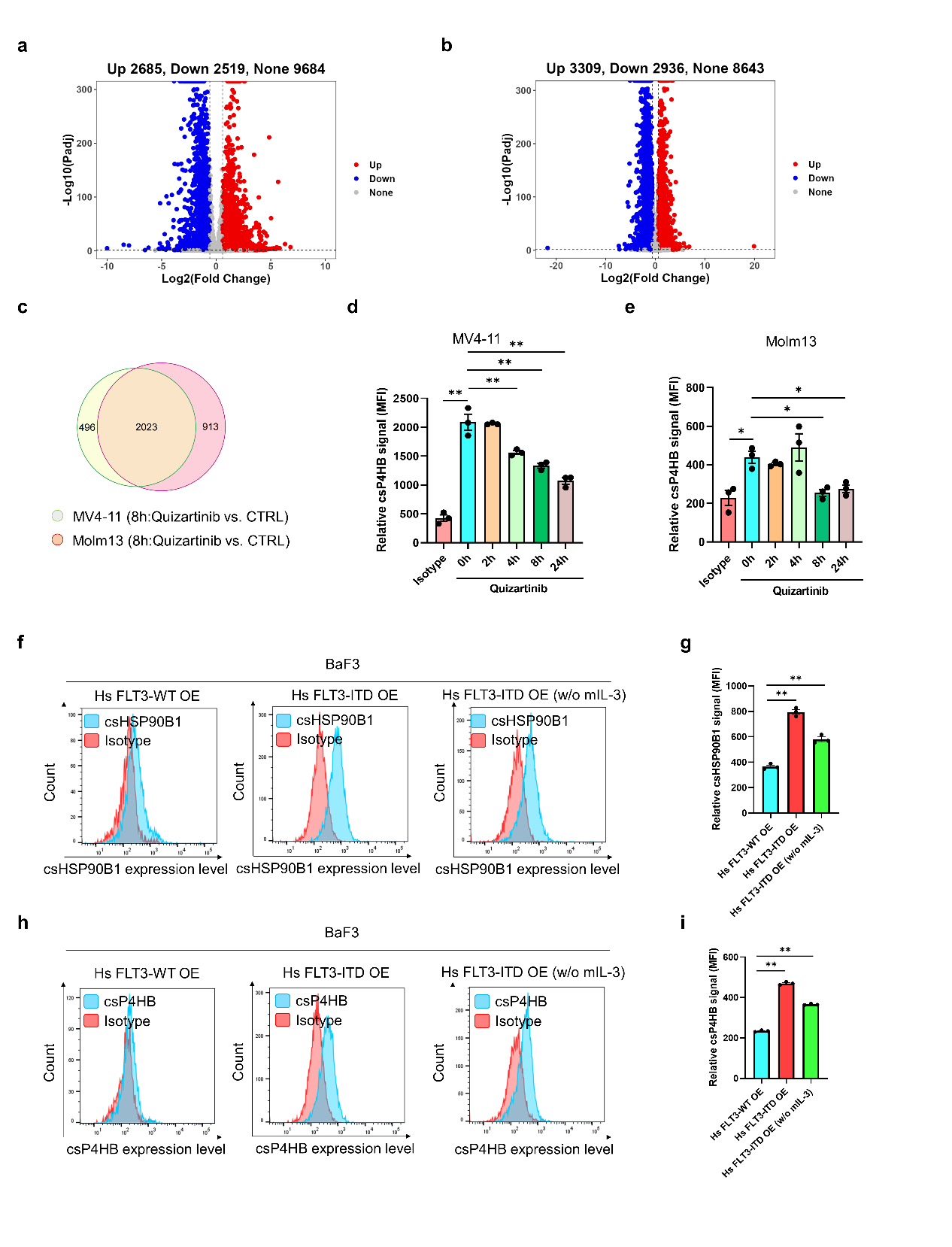


Figure S6. ER stress-mediated IRE1α-SRC signaling axis as a mechanistic driver of chaperone surface translocation in AML living cells. a) Flow cytometry analysis showing that IRE1α inhibitor treatment (1 μM, 24 h) decreased cell-surface HSP90B1 expression in living MV4-11. b) Quantification of cell-surface HSP90B1 expression levels in living MV4-11 cells from (a). Data represent as mean ± SEM of fluorescence intensity (MFI), *p*-values are calculated using Student’s *t-*test, *n*=3, ***p* < 0.01. c) Flow cytometry analysis showing that IRE1α inhibitor treatment (1 μM, 24 h) decreased cell-surface HSP90B1 expression in living Molm13. d) Quantification of cell-surface HSP90B1 expression levels in living Molm13 cells from (c). Data represent as mean ± SEM of fluorescence intensity (MFI), *p*-values are calculated using Student’s *t-*test, *n*=3, ***p* < 0.01. e) Flow cytometry analysis showing that SRC inhibitor treatment (20 μM, 24 h) decreased cell-surface HSP90B1 expression in living MV4-11. f) Quantification of cell-surface HSP90B1 expression levels in living MV4-11 cells from (e). Data represent as mean ± SEM of fluorescence intensity (MFI), *p*-values are calculated using Student’s *t-*test, *n*=3, ***p* < 0.01. g) Flow cytometry analysis showing that SRC inhibitor treatment (20 μM, 24 h) decreased cell-surface HSP90B1 expression in living Molm13. h) Quantification of cell-surface HSP90B1 expression levels in living Molm13 cells from (g). Data represent as mean ± SEM of fluorescence intensity (MFI), *p*-values are calculated using Student’s *t-*test, *n*=3, ***p* < 0.01.


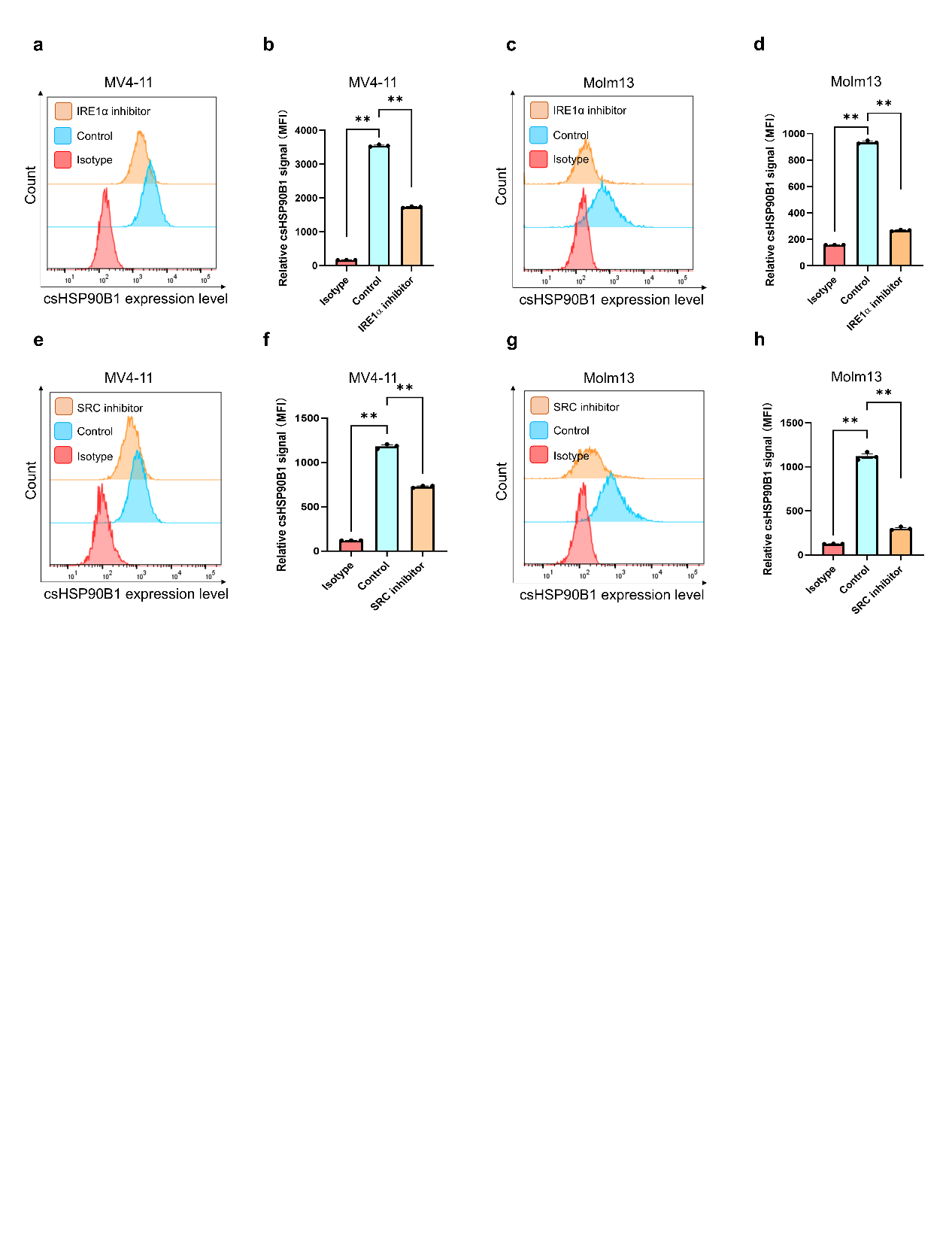


Figure S7. Single gene GSEA functional enrichment analysis based on the gene expression profiles correlated with *P4HB* or *HSP90B1*. a, b) Single gene GSEA functional enrichment analysis based on the gene expression profiles correlated with *P4HB* (a) or *HSP90B1* (b) in these RNA-seq datasets of 131 FLT3-ITD^+^ AML patients. c, d) GSEA plots of the main enriched GO functional gene sets of *P4HB* (c) or *HSP90B1* (d) with NES>0 for these RNA-seq datasets of 131 FLT3-ITD^+^ AML patients. Detection is based on the enriched genes for each GO terms.


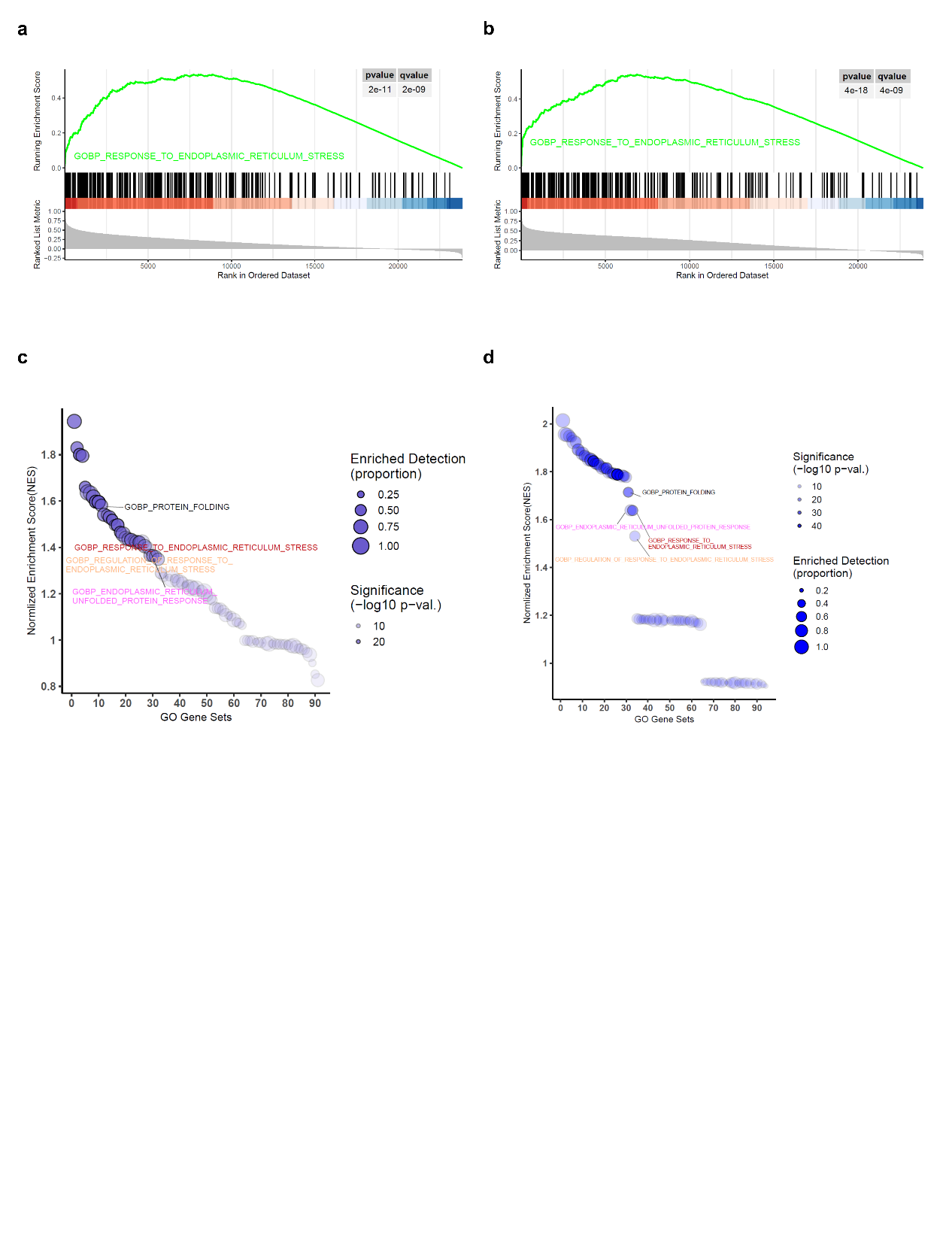


Figure S8. Cell-surface chaperone HSP90B1 is undetectable in subpopulations from healthy bone marrow cells. a) Flow cytometry analysis of csHSP90B1 expression in CD3⁺ T cells. b) Flow cytometry analysis of csHSP90B1 expression in CD19⁺ B cells. c) Flow cytometry analysis of csHSP90B1 expression in CD56⁺ natural killer cells. d) Flow cytometry analysis of csHSP90B1 expression in CD34⁺ hematopoietic stem/progenitor cells (HSPCs).


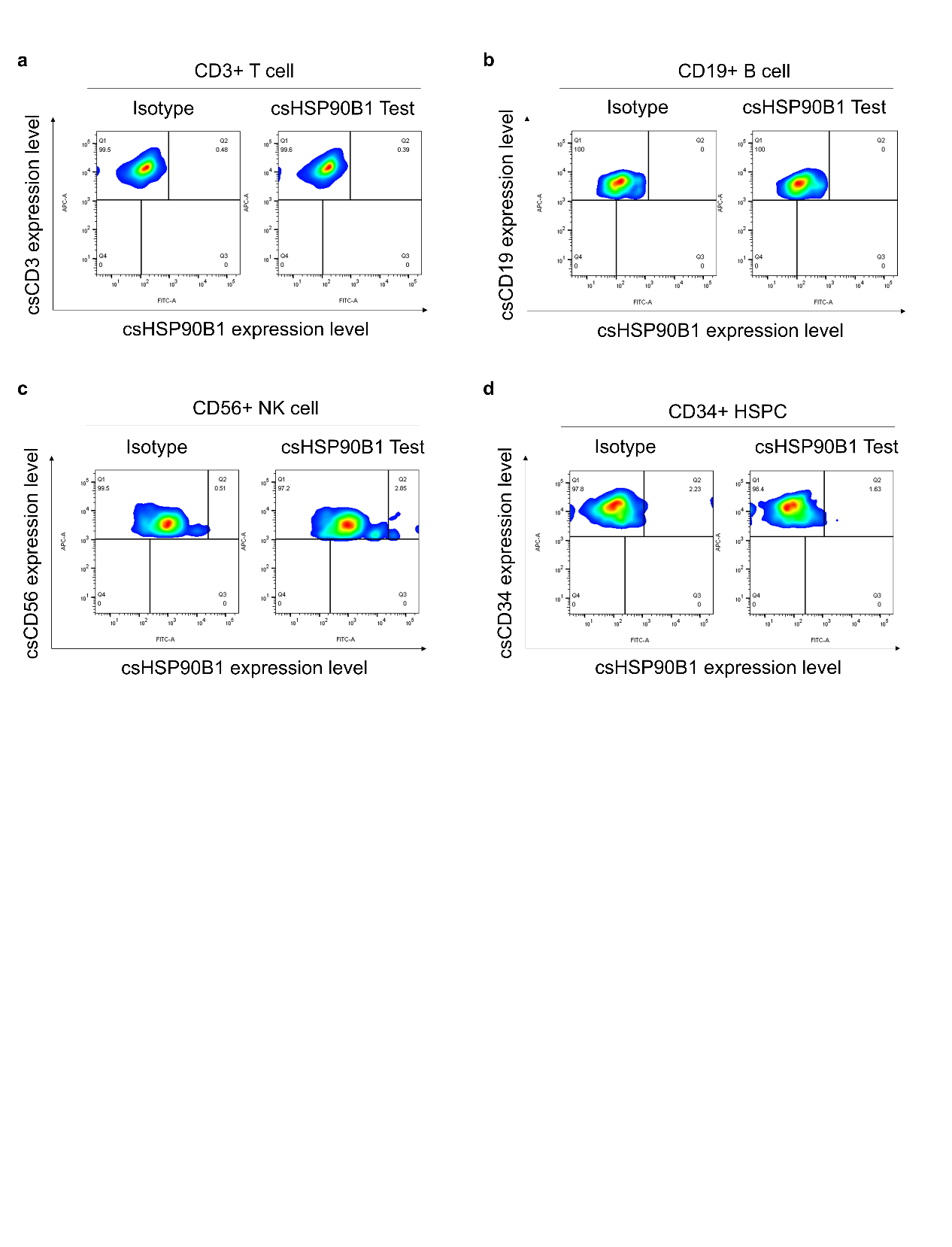


Figure S9. Cell-surface chaperone HSP90B1 (csHSP90B1) and P4HB (csP4HB) are undetectable in murine tissues and organs. a) Flow cytometry analysis of csHSP90B1 expression in different tissues or organs from mouse. b) Flow cytometry analysis of csP4HB expression in different tissues or organs from mouse.


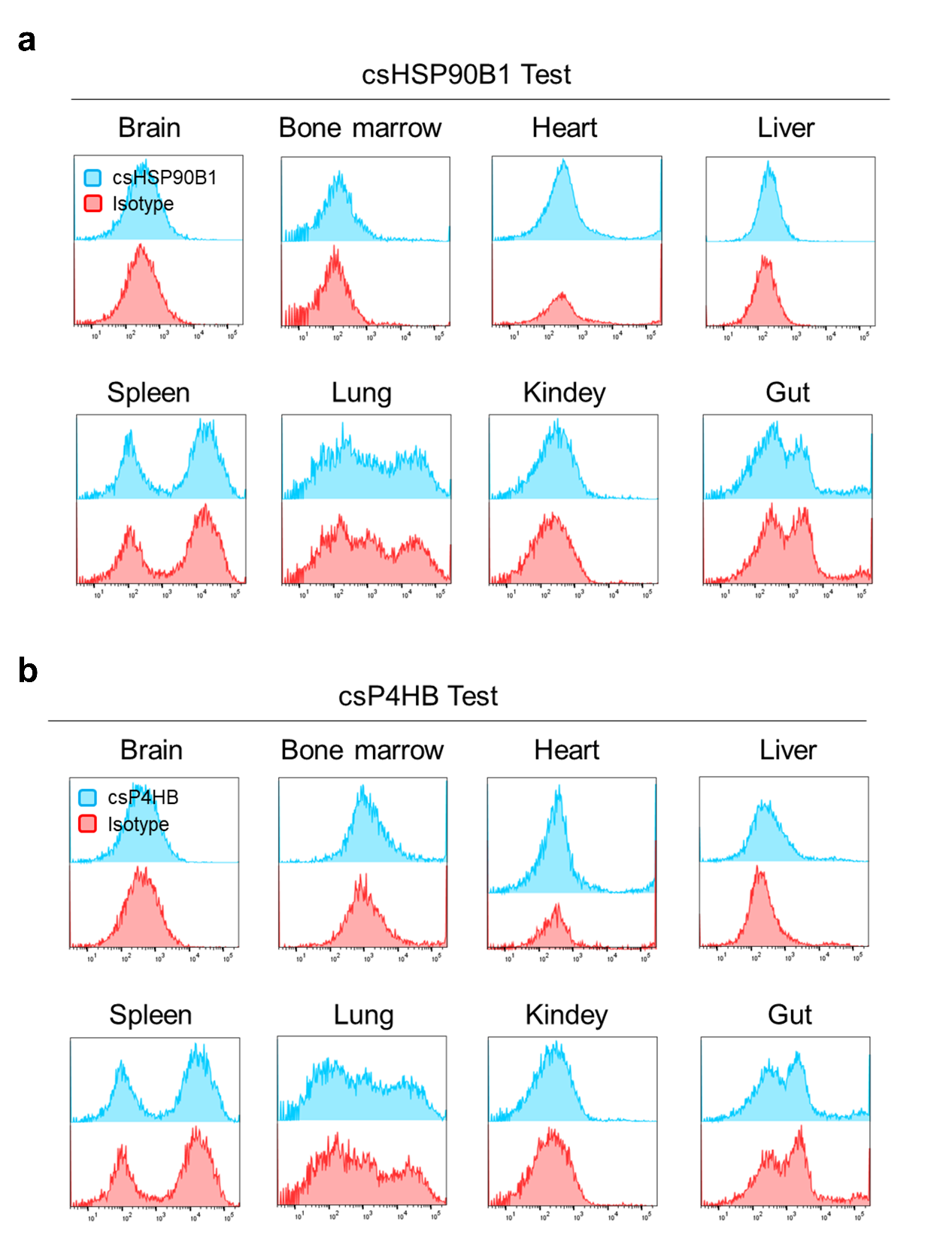


Figure S10. Single-cell transcriptional analyses of the bone marrow cell samples from healthy donors. a, b) UMAP visualization of bone marrow (BM) cells from four healthy donors, showing 14 clusters (a), with 12 annotated cell subpopulations presented (b). c) Split bar plots illustrating the cellular composition and distribution across each sample. d) Violin plots displaying the expression of a representative cell marker for each cell type in normal BM samples.


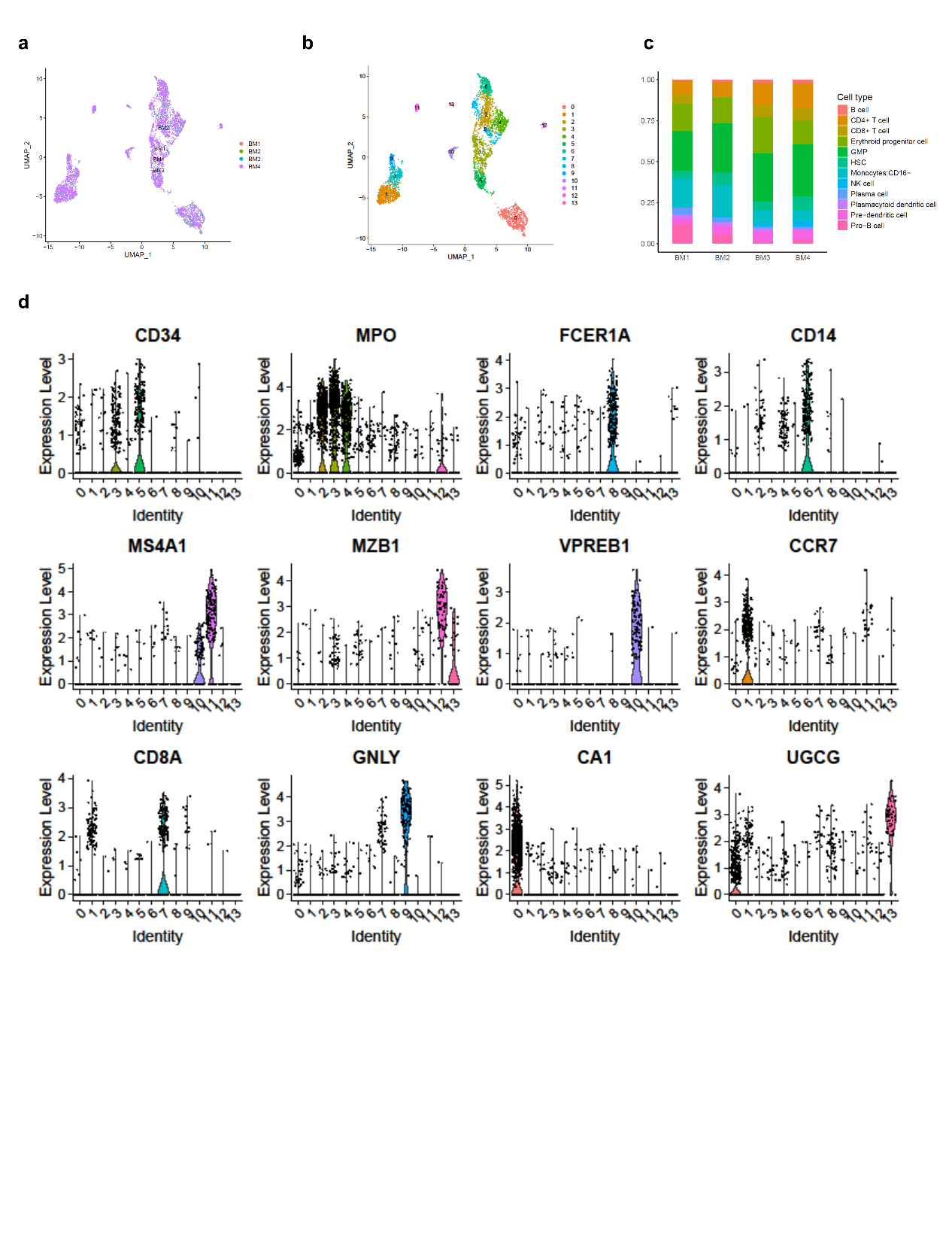


Figure S11. Single-cell transcriptional analyses of the bone marrow sample from FLT3-ITD^+^ AML patients. a, b) UMAP visualization of clustered bone marrow (BM) cells from four AML patients, with distinct clusters represented by different colors (a), and nine main clusters identified as eight cell types (b). c) Split bar plots illustrating the cellular composition and distribution across each sample. d, e, f) Individual cell annotations for three AML patients carrying the FLT3-ITD^+^ mutation. g) Violin plots depicting the expression of representative cell markers for each cell type in the diseased BM samples.


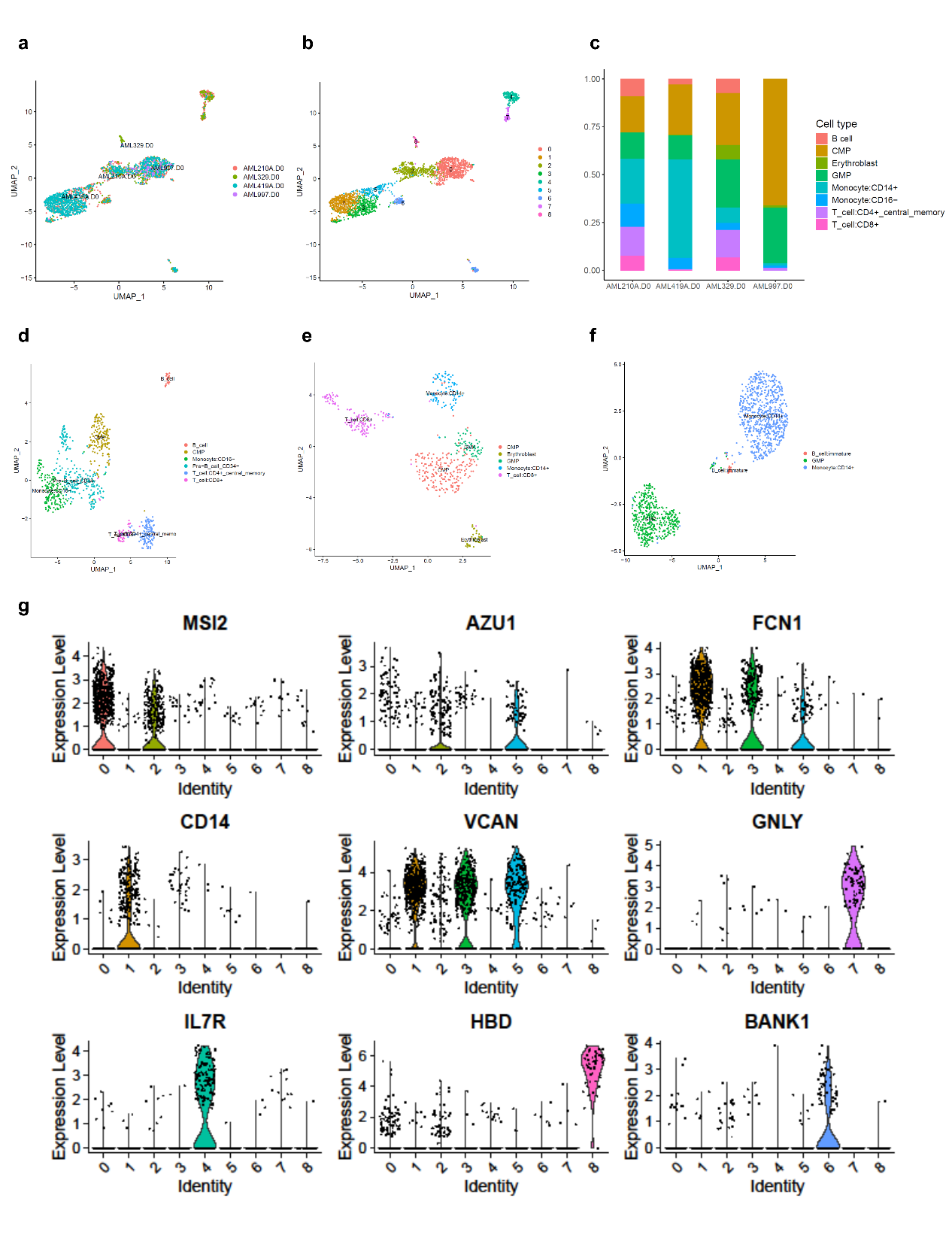


Figure S12. The expression of chaperones including *HSP90B1* and *P4HB* in myeloid cells and non-myeloid cells from the bone marrow samples of healthy donors and FLT3-ITD^+^ AML patients. a, b) Expression data of *HSP90B1* (a) and *P4HB* (b) in cell clusters classified as potential myeloid cells and non-myeloid cells in bone marrows from 4 cases of FLT3-ITD^+^ AML patients. c, d) Expression data of *HSP90B1* (c) and *P4HB* (d) in cell clusters classified as potential myeloid cells and non-myeloid cells in bone marrows from 4 cases of healthy donors. In AML patient samples, the expression levels of *HSP90B1* and *P4HB* were significantly higher (fold change ≥1.5, adjusted *p* < 0.05) in malignant myeloid cells than in non-myeloid cells, whereas no such upregulations were observed in healthy samples.


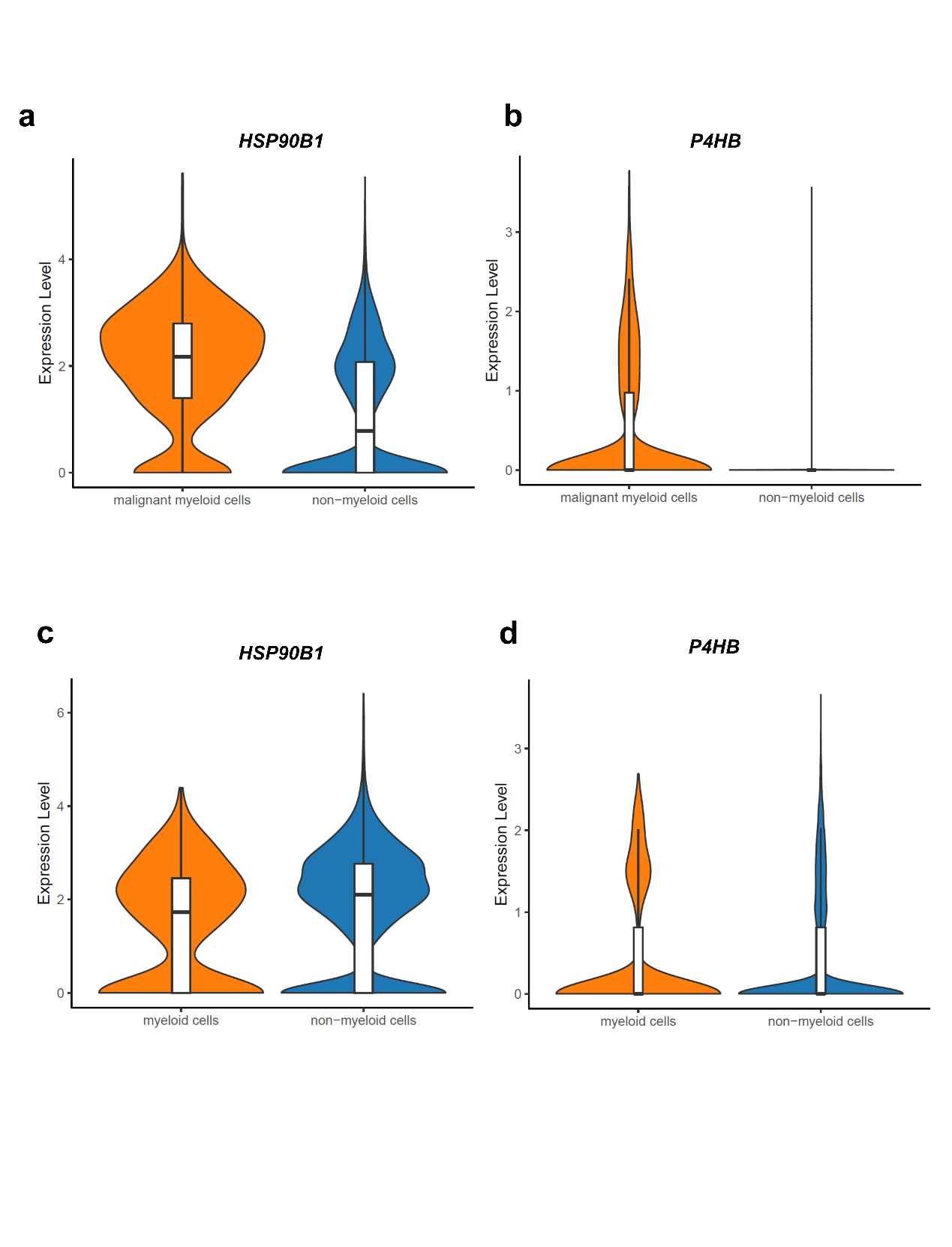


Figure S13. CAR-NK cells show no detectable csHSP90B1 and no cytotoxicity against effector cell but display serious toxicity against target cells. a, b) Validation of extracellular exposure of translocated HSP90B1 chaperone proteins tested by flow cytometry on viable MV4-11 (a) and Molm13 (b) leukemic cells using His-tagged scFvs derived from chimeric antigen receptor (CAR) constructs. c) Flow cytometry analysis of csHSP90B1 expression on NK92 cells and CAR-transduced NK92 cells. d) Enhanced apoptosis observed in MV4-11 cells co-cultured with csHSP90B1 CAR NK92 cells compared with control NK92 cells at an E/T ratio of 2:1 for 12 h. e) Immunofluorescence images showing csHSP90B1 expression in non-permeabilized THP-1 cells. f) Flow cytometry analysis of csHSP90B1 expression in living THP-1 cells. g) Relative apoptosis percentage of THP-1 cells after 6 h of co-culture with csHSP90B1 CAR NK92 cells at an E/T ratio of 2:1. Data represent as mean ± SEM, *p*-values are calculated using Student’s *t-*test, *n*=3, ***p* < 0.01. h, i) Flow cytometry analysis of csHSP90B1 expression in living AML cells with c-Kit mutations including SKNO-1 (h) and Kasumi-1 (i) cell lines.


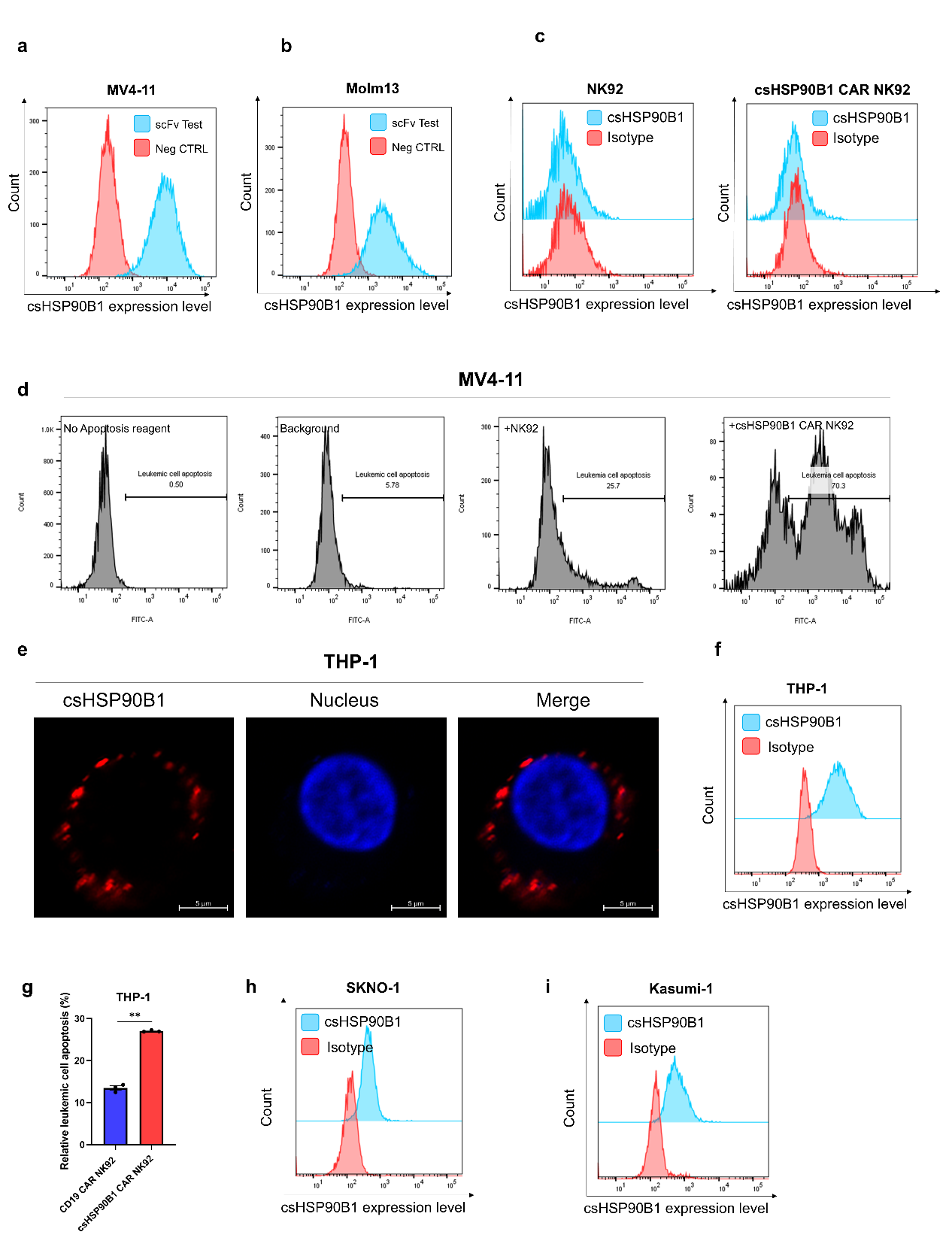


Figure S14. IFN-γ stimulation increases cell-surface PD-L1 protein expression on the target cells during target cell apoptosis mediated by csHSP90B1 CAR NK92 cells. a) Flow cytometry analysis showing that IFN-γ stimulation (50 ng/ml for 24 h) increased PD-L1 expression on MV4-11 cell surface. b) Quantification of cell-surface PD-L1 expression levels in living MV4-11 cells from (a). Data represent as mean ± SEM of fluorescence intensity (MFI), *p*-values are calculated using Student’s *t-*test, *n*=3, ***p* < 0.01. c) Flow cytometry analysis showing that IFN-γ stimulation (50 ng/ml for 24 h) enhanced PD-L1 expression on Molm13 cell surface. d) Quantification of cell-surface PD-L1 expression levels in living Molm13 cells from (c). Data represent as mean ± SEM of fluorescence intensity (MFI), *p*-values are calculated using Student’s *t-*test, *n*=3, ***p* < 0.01. e) Flow cytometry analysis of PD-L1 expression levels on the surface of living MV4-11 cells without or with csHSP90B1 CAR NK92 co-culture at different E/T ratios for 24 h. f) Relative PD-L1 expression levels on the surface of living MV4-11 cells without or with csHSP90B1 CAR NK92 co-culture at different E/T ratios for 24 h, as quantified from (e). Data represent as mean ± SEM of fluorescence intensity (MFI), *p*-values are calculated using Student’s *t-*test, *n=*3, ***p* < 0.01. g) Flow cytometry analysis of PD-L1 expression levels on the surface of living Molm13 cells without or with csHSP90B1 CAR NK92 co-culture at different E/T ratios for 24 h. h) Relative PD-L1 expression levels on the surface of living Molm13 cells without or with csHSP90B1 CAR NK92 co-culture at different E/T ratios for 24 h, as quantified from (g). Data represent as mean ± SEM of fluorescence intensity (MFI), *p*-values are calculated using Student’s *t-*test, *n*=3, ***p* < 0.01.


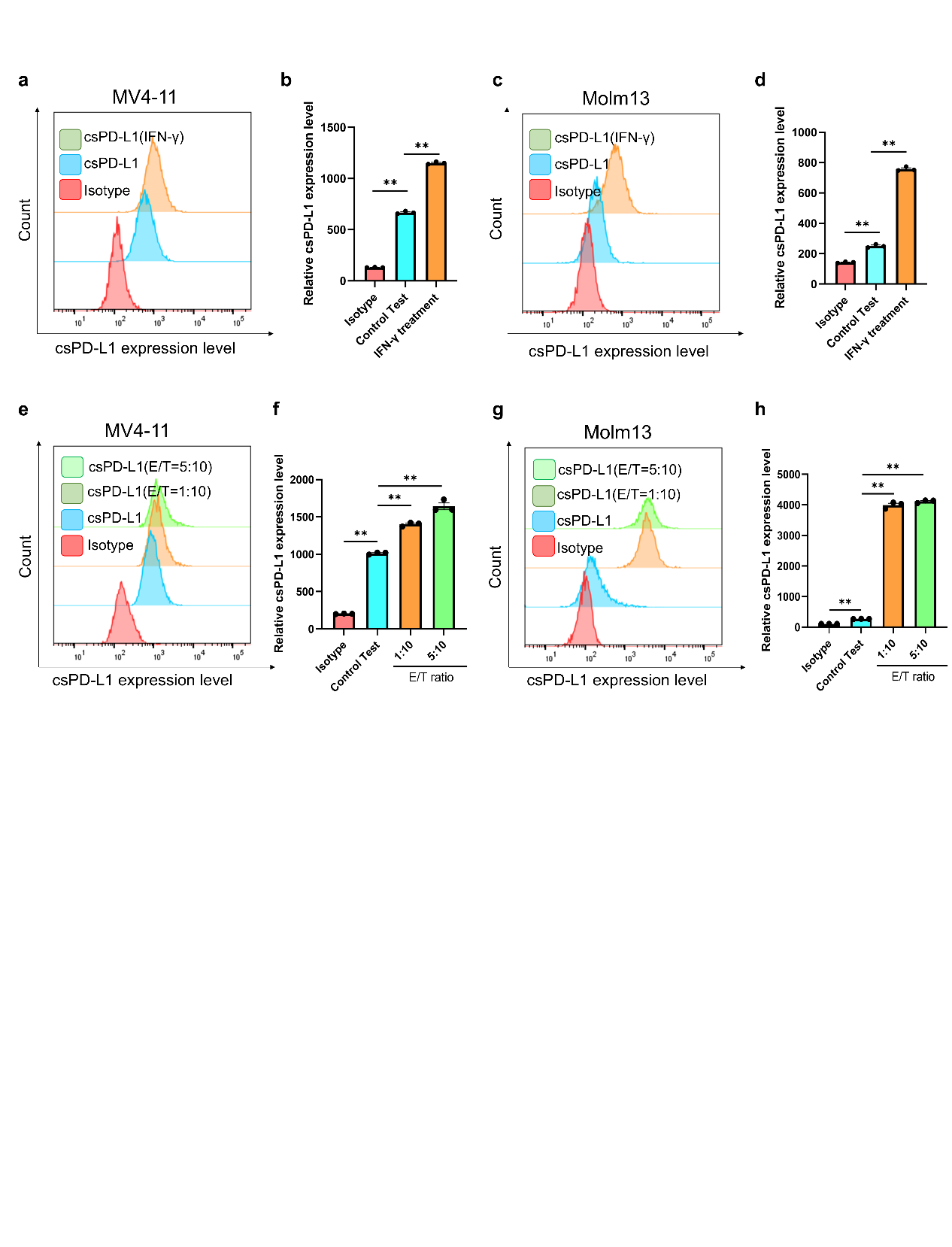


Figure S15. The csHSP90B1 CAR cell preliminary toxicity evaluation in mice. a) Experimental design for preliminary toxicity evaluation of csHSP90B1 CAR NK92 cells using a mouse xenograft model. b) Body weight assessment of xenografted mice during injections of four doses of csHSP90B1 CAR-NK92 cells. Data represent as mean ± SEM. *n*=3 in each group. c) Body weight assessment of xenografted mice one week after the final dose of csHSP90B1 CAR-NK92 cell treatment. Data represent as mean ± SEM, *p*-values are calculated using Student’s *t-*test, *n*=3, ns, not significant. d) Survival ratio of xenografted mice one week after the final dose of csHSP90B1 CAR-NK92 cell injection. *n*=3 in each group.


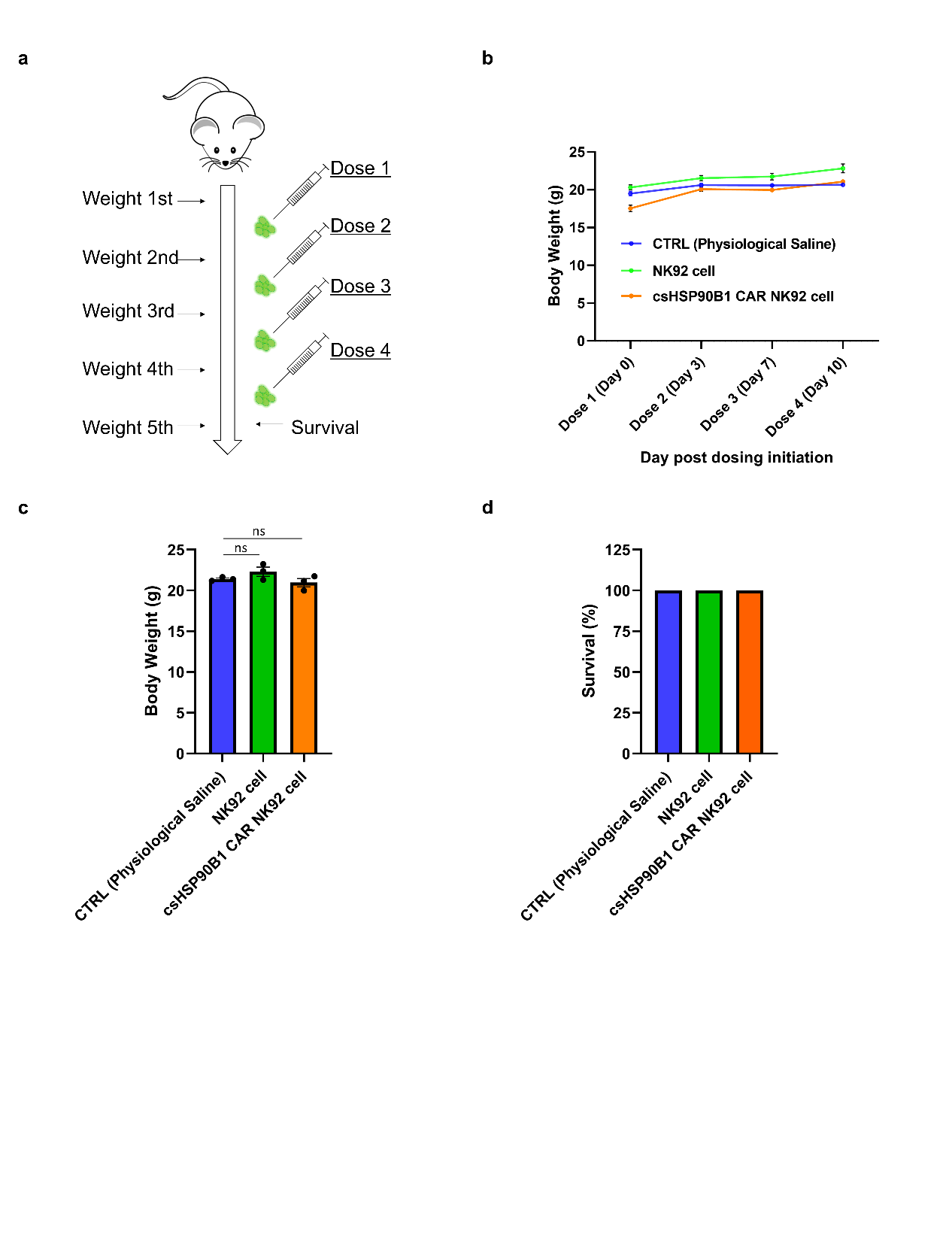


Figure S16. Pairwise sequence alignment of human and mouse HSP90B1 protein sequences. The protein sequences of human HSP90B1 (Accession: NP_003290.1) and mouse hsp90b1 (Accession: NP_035761.1) were aligned using the BLASTP algorithm. Identical amino acid residues are highlighted with a background color, while non-identical residues are shown without coloring. The two sequences share an overall identity of 96.64%.


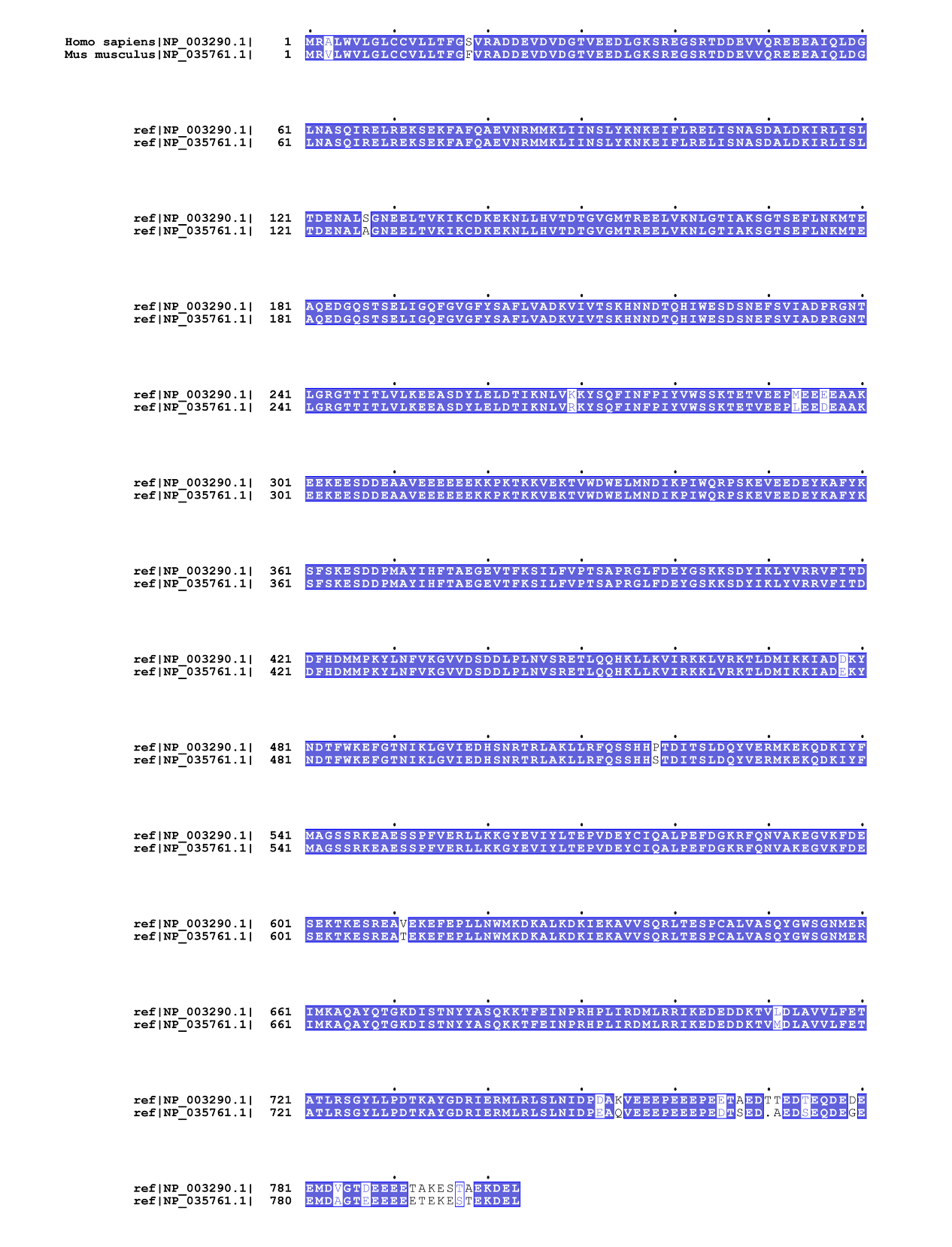


List of Movies

Movie S1. Process of CAR-NK cell-mediated target cell apoptosis (AML cell line).

Movie S2. Process of CAR-NK cell-mediated target cell apoptosis (AML primary cells).

List of Datasets

Dataset S1. List of proteins subjected to mass spectrometry.

Dataset S2. Comparisons of proteins analyzed using mass spectrometry.

Dataset S3. List of functional combined GO sets.
